# Hematopoietic transcription factor GFI1 promotes anchorage independence by sustaining ERK activity in cancer cells

**DOI:** 10.1172/JCI149551

**Published:** 2022-09-01

**Authors:** Hao Wang, Zhenzhen Lin, Zhe Nian, Wei Zhang, Wenxu Liu, Fei Yan, Zengtuan Xiao, Xia Wang, Zhenfa Zhang, Zhenyi Ma, Zhe Liu

**Affiliations:** 1 State Key Laboratory of Experimental Hematology, Haihe Laboratory of Cell Ecosystem, The Province and Ministry Co-sponsored Collaborative Innovation Center for Medical Epigenetics, Key Laboratory of Immune Microenvironment and Disease of the Ministry of Education, Department of Immunology, School of Basic Medical Sciences, Tianjin Medical University, Tianjin, China.; 2 Department of Lung Cancer Center and; 3 Department of Gastroenterology, Tianjin Medical University Cancer Institute and Hospital, Tianjin, China.; 4 Department of Cell Biology, School of Basic Medical Sciences, Hangzhou Normal University, Hangzhou, China.; 5 Collaborative Innovation Center for Cancer Personalized Medicine, Nanjing Medical University, Nanjing, China.

**Keywords:** Cell Biology, Oncology, Apoptosis survival pathways, Cancer, Cell migration/adhesion

## Abstract

The switch from anchorage-dependent to anchorage-independent growth is essential for epithelial metastasis. The underlying mechanism, however, is not fully understood. In this study, we identified growth factor independent-1 (GFI1), a transcription factor that drives the transition from adherent endothelial cells to suspended hematopoietic cells during hematopoiesis, as a critical regulator of anchorage independence in lung cancer cells. GFI1 elevated the numbers of circulating and lung-infiltrating tumor cells in xenograft models and predicted poor prognosis of patients with lung cancer. Mechanistically, GFI1 inhibited the expression of multiple adhesion molecules and facilitated substrate detachment. Concomitantly, GFI1 reconfigured the chromatin structure of the *RASGRP2* gene and increased its expression, causing Rap1 activation and subsequent sustained ERK activation upon detachment, and this led to ERK signaling dependency in tumor cells. Our studies unveiled a mechanism by which carcinoma cells hijacked a hematopoietic factor to gain anchorage independence and suggested that the intervention of ERK signaling may suppress metastasis and improve the therapeutic outcome of patients with GFI1-positive lung cancer.

## Graphical abstract

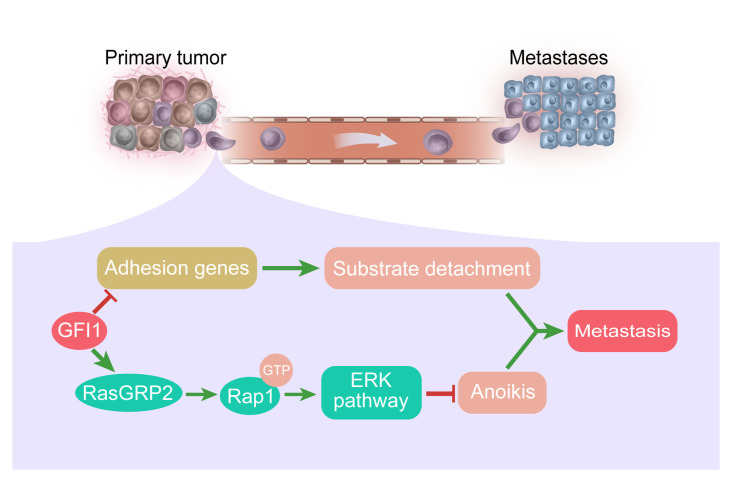

## Introduction

Epithelial cells receive growth and survival stimuli through attachment to the extracellular matrix (ECM) (1). The loss of attachment to ECM triggers apoptosis, commonly referred to as anoikis (2, 3). Overcoming the addiction to ECM-induced signals is required for anchorage-independent growth, characteristic of transformation and malignancy (4). A better understanding of the mechanisms governing the transition from anchorage dependence to anchorage independence is instrumental for novel therapeutic strategies that target metastatic cells detached from the ECM.

The transition from anchorage dependence to anchorage independence during malignant transformation is associated with global changes in transcriptional programs. For example, altered expression of integrins, cadherins, and apoptotic genes has been linked to every step of the metastatic cascades in many types of cancer cells (5–7). A large number of studies have shown that epithelial-mesenchymal transition (EMT), a cellular program used by embryos to enhance cell motility, is hijacked by cancer cells to disseminate from their primary site (8–10). EMT transcription factors (ZEB1, Snail1, Slug, Twist) can downregulate genes involved in cell-cell and cell-substrate interactions and promote resistance to anoikis (11, 12). A longstanding question, however, is whether other cellular programs that control normal physiological processes are also involved in the regulation of anchorage independence of cancer cells.

In this study, we identified GFI1, a hematopoietic transcription factor known to drive the transition from adherent endothelial cells to suspended hematopoietic cells during early hematopoiesis, as a key factor endowing epithelial cancer cells with anchorage independence. We explored the underlying mechanism by which GFI1 regulates the transcriptional program, promoting detachment, anoikis resistance, and metastasis, and we evaluated its clinical implications in predicting sensitivity to ERK signaling inhibitors in cancer treatment.

## Results

### GFI1 expression correlates with poor prognosis of patients with lung cancer.

**Figure 1:**
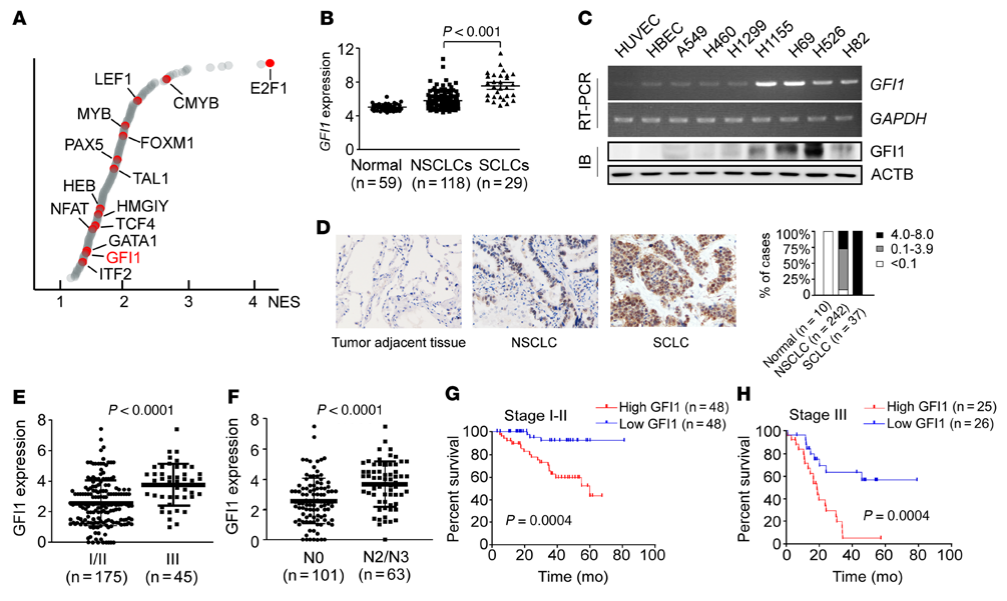
GFI1 is expressed in lung cancer cells and predicts poor prognosis. (**A**) Transcription factor binding motif enrichment in 29 human SCLC lines versus 118 NSCLC lines generated by Minna et al. (**B**) *GFI1* expression in various lung cell types were screened in the Minna et al. study’s transcriptional profiles. *GFI1* expression was significantly higher in SCLCs than in NSCLCs and was enhanced in both groups compared with normal epithelium. (**C**) RT-PCR and immunoblot of GFI1 expression in the indicated cell lines. See complete unedited blots in the supplemental material. (**D**) IHC staining with anti-GFI1 antibody was performed on 10 normal lung tissues adjacent to tumors and 242 NSCLC and 37 SCLC specimens. Scale bars: 20 μm. The frequency of samples with no (0), low (0.1–3.9), or high (4.0–8.0) GFI1 staining stratified by IHC-defined lung cancer subtype. (**E**) High GFI1 expression level correlated with stage III disease in NSCLC. (**F**) High GFI1 expression level correlated with distant lymph node metastasis in NSCLC. (**G**) Kaplan-Meier survival rates for 96 patients with stage I–II NSCLC disease with low versus high GFI1 expression were compared. Median value of GFI1 expression was used as the cutoff. (**H**) Kaplan-Meier survival rates for 51 patients with stage III NSCLC disease with low versus high GFI1 expression were compared. Median value of GFI1 expression was used as the cutoff.

Small cell lung cancer (SCLC) is a highly aggressive metastatic cancer. It is clinically characterized by early dissemination and rapid tumor growth. Unlike non-SCLCs (NSCLCs), SCLC cells survive as floating clusters in pleural fluid in vivo and grow as suspension in vitro, exhibiting high resistance to anoikis (13). To identify the key transcription factors that regulate anchorage independence, we analyzed transcriptional profiles of 29 human SCLC lines and 118 human NSCLC lines generated by Minna and colleagues (14, 15) for transcription factor binding motif enrichment. We identified 190 transcription factors as significantly active in SCLCs compared with NSCLCs (Figure 1A and Supplemental Table 1; supplemental material available online with this article; https://doi.org/10.1172/JCI149551DS1). As expected, the binding motifs of E2F1, a transcription factor that can be activated by the loss of the retinoblastoma susceptibility gene (RB1), were enriched in SCLCs (Figure 1A). RB inactivation has been identified in up to 90% of SCLCs and is considered as an initiating molecular event of SCLCs (16). Among the 190 SCLC-active transcription factors, 38 transcription factors are involved in neural or endocrine development, for example, ZIC2, NGFIC, EGR3, and LHX3, consistent with the finding that SCLCs express a neuroendocrine program (17). Fourteen transcription factors are involved in hematopoietic development, in which GFI1, a zinc finger protein, is of particular interest, since it is normally expressed at the very early onset of hematopoietic differentiation and plays a critical role in turning adherent hemogenic endothelial cells into suspended hematopoietic progenitors (18). We therefore explored the possibility of GFI1 in mediating anchorage independence in metastatic epithelial cancer cells.

We first studied GFI1 expression in established lung cancer cell lines. We interrogated again the Minna et al. study’s transcriptional profiles for *GFI1* expression. In this analysis, we included 59 cell lines derived from normal lung epithelium besides the 118 NSCLC lines and 29 SCLC lines. *GFI1* expression was significantly higher in SCLCs than in NSCLCs, and each group was increased compared with normal epithelium (Figure 1B). Reverse transcription–PCR (RT-PCR) and immunoblot in a subset of selected cells confirmed the higher level of GFI1 expression in SCLC lines (H526, H69, and H82) and in neuroendocrine NSCLC line H1155, all of which proliferate as clusters in suspension in vitro, compared with primary HUVECs, an immortalized line of human bronchial epithelial cells (HBECs), and lung adenocarcinoma cell lines (A549 and H460), which proliferate as adherent single cells (Figure 1C). Thus, GFI1 expression was enhanced in suspended cancer cells and may be associated with the gain of anchorage independence.

We next examined GFI1 expression in tissue sections of 242 primary NSCLCs, 37 primary SCLCs, and 10 tumor-adjacent normal lung tissues using IHC. Epithelial cells in tumor-adjacent lung tissues did not express GFI1 (Figure 1D). Both NSCLC and SCLC expressed GFI1 (Figure 1D). Quantification of staining based on the intensity of GFI1 nuclear staining and the percentage of GFI1-positive tumor cells revealed higher expression of GFI1 in SCLCs than in NSCLCs (Figure 1D), which matched the GFI1 expression pattern detected in the cell lines. Consistently, analysis of single-cell RNA-Seq data sets of human lung cancers generated by Rudin and colleagues (19) also revealed higher expression of *GFI1* in SCLCs than in NSCLCs: all 4 primary SCLCs contained GFI1-expressing cancer cells, whereas only 8 out of 14 primary NSCLCs contained *GFI1*-expressing cancer cells (Supplemental Figure 1A). In addition, gene set enrichment analysis (GSEA) revealed that *GFI1* was associated with the genes involved in negative regulation of anoikis (Supplemental Figure 1B and Supplemental Table 2).

To assess the prognostic significance of GFI1, we examined the expression levels in resected NSCLC tumors from patients with known clinical outcomes. Increased expression of GFI1 was associated with tumor stage and lymphatic metastasis (Figure 1, E and F; unpaired *t* test, *P* < 0.0001). In addition, patients with early-stage disease (stage I–II, *n* = 96) and tumors of low GFI1 expression levels (*n* = 48) had strikingly longer overall survival times than those with tumors of high GFI1 expression levels (*n* = 48) (median survival: high GFI1 expression 60 months; low GFI1 expression undefined; *P* = 0.0004). Of 51 patients with stage III disease, patients with low GFI1 expression (*n* = 25) had better overall survival than high GFI1 expression (*n* = 26) (median survival: high GFI1 expression 18.79 months; low GFI1 expression undefined; *P* = 0.0004). The survival rate of the high GFI1 expressors with stage III disease was poor and similar to that for extensive-stage disease SCLC. In a Kaplan-Meier model, GFI1 protein expression was a strong predictor of survival rates in patients with stage I or II lung cancer (hazard ratio 4.573, 95% CI 1.98–10.56, Figure 1G) and in those with stage III disease (hazard ratio 3.83, 95% CI 1.814–8.085, Figure 1H). These results indicate that the hematopoietic lineage protein GFI1 was frequently expressed in lung cancers, and its high expression level correlated with distant lymph node metastasis and extremely poor survival rates in human lung cancer.

To generalize this finding, we analyzed GFI1 expression in other types of cancer. GFI1 was also frequently expressed in human primary breast and ovarian cancer cells (Supplemental Figure 2, A and B). High expression of GFI1 was also associated with poor prognosis for patients with breast cancer and ovarian cancer (Supplemental Figure 2, C and D). These results suggest that GFI1 may be used as a broad prognostic marker for multiple types of cancer.

### GFI1 promotes the detachment of cancer cells from the substrate.

**Figure 2:**
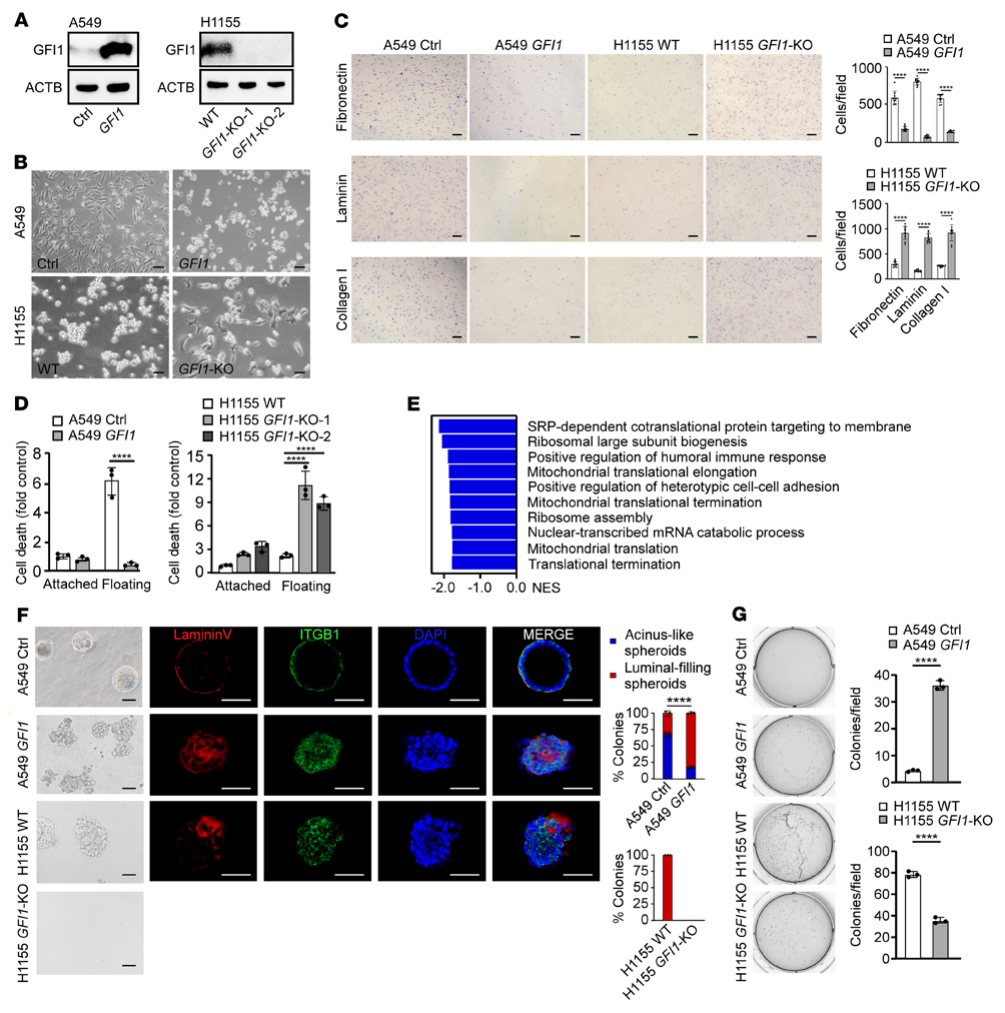
GFI1 promotes cell detachment and anchorage independence. (**A**) Immunoblot showing expression of GFI1 and ACTB. See complete unedited blots in the supplemental material. (**B**) Phase-contrast micrographs of *GFI1*-expressing A549 and *GFI1*-KO H1155 cells. Scale bars: 40 μm. (**C**) *GFI1*-expressing A549 cells or *GFI1*-KO H1155 cells were plated on fibronectin-coated, laminin332-coated, or collagen I*–*coated plates. After 15 minutes, attached cells were counted. Scale bars: 200 μm. Bar graph shows the number of adherent cells. Mean ± SD represents 10 visualized areas in 1 experiment. Three independent experiments were performed. ****P < 0.0001 (unpaired 2-tailed Student’s *t* test). (**D**) The cell death of *GFI1*-expressing A549 cells and *GFI1*-KO H1155 cells was assessed after 24 hours under attached or floating condition. Mean ± SD represents 3 replicates in 1 experiment. Three independent experiments were performed. ****P < 0.0001 (1-way ANOVA test with post hoc contrasts by Tukey’s test). (**E**) A549 and *GFI1*-expressing A549 cells were forced into suspension for 48 hours and transcriptional profiles were measured using RNA-Seq. Gene set enrichment analysis showing the top 10 downregulated pathways in *GFI1*-expressing cells versus control A549 cells. (**F**) A549, *GFI1*-expressing A549, H1155, and *GFI1*-KO H1155 cells were cultured in Matrigel for 8 days. Confocal midpoint slices of acinus stained for ITGB1, laminin V, and DAPI are shown. Colonies greater than 50 μm in diagram were counted. Scale bars: 50 μm. Mean ± SD represents 10 visualized areas in 1 experiment. Three independent experiments were performed. Data shown as mean ± SEM. ****P < 0.0001 (unpaired 2-tailed Student’s *t* test). (**G**) Indicated cells were allowed to grow in soft agar for 2 weeks, and colonies were counted. Data shown as mean ± SD for a representative experiment performed in triplicate. Three independent experiments were performed.

To explore the function of GFI1 in malignant progression, we assessed the effects of GFI1 overexpression and deletion. We expressed GFI1 in GFI1-negative A549 cells using a lentiviral system and deleted GFI1 in GFI1-positive H1155 cells using a CRISPR/Cas9 system (Figure 2A) and subsequently analyzed the alterations in cellular behaviors. Consistent with its documented function as a regulator of the transition from adherent endothelium cells to round suspended hematopoietic cells during early hematopoiesis, GFI1 overexpression induced A549 cells to undergo changes from adherent and spread-out morphologies to less adherent and round morphologies (Figure 2B). This effect of GFI1 overexpression is not cell type specific because GFI1 overexpression caused similar morphological changes in other lung cancer cell lines, including H460 and H1299, breast cancer cell lines MCF7 and MDA-MB-231, ovarian cancer cell line SKOV3, and colorectal cancer cell line HT-29 (Figure 2B and Supplemental Figure 3A). Conversely, neuroendocrine NSCLC cells H1155, which normally proliferate as clusters in suspension, became single cells with substrate adherence after *GFI1* deletion (Figure 2B).

We then tested the effect of GFI1 overexpression on adherence of lung cancer cells to fibronectin, laminin, and collagen, 3 major ECM components in lung tumor stroma. We found GFI1 overexpression dramatically decreased the number of A549 and H460 cells that adhered to all 3 ECM components, whereas GFI1 deletion increased the adhesion of H1155 cells (Figure 2C and Supplemental Figure 3B). Thus, GFI1 facilitated cancer cell detachment from substrate. However, GFI1 overexpression repressed migration and invasion of A549 and H460 cells, evaluated by wound healing and Boyden chamber assays (Supplemental Figure 4, A and B). GFI1 was not able to increase invasive growth in 3D dense gel composed of collagen I and Matrigel (Supplemental Figure 4C). GFI1 overexpression had no or negative effect on proliferation of H460 and A549 cells, whereas deletion of GFI1 in H1155 cells also repressed proliferation, evaluated by 5-BrdU incorporation, indicating that the role of GFI1 in proliferation was context dependent (Supplemental Figure 5). The cell viability was tested before these assays (Supplemental Figure 6).

### GFI1 promotes anchorage independence.

While tumor cells display some degree of anchorage independence, their detachment from the ECM can promote cell death. We therefore asked whether GFI1 induction of substrate detachment may lead to anoikis. We overexpressed GFI1 in anoikis-susceptible cells, including HUVECs and HBECs, lung cancer cells (A549, H460, H1299), ovarian cancer cells (SKOV3, OVCA432), liver cancer cells (HepG2, Hep3B), breast cancer cells (MDA-MB-231, MCF7), and colorectal cancer cells (HT-29, HCT8), and subsequently forced the cells into suspension by culturing them on low-attachment plates. Overexpression of GFI1 suppressed cell death in all the suspended cells we examined (Figure 2D and Supplemental Figure 7), while deletion of GFI1 increased cell death in H1155 cells (Figure 2D). Consistently, a significantly lower level of cleaved PARP was detected in suspended GFI1-expressing cells (A549-GFI1, H460-GFI1, H1155) than in suspended GFI1-negative cells (A549, H460, H1155-*GFI1*-KO). Caspase-3 was not involved in this suspension-induced cell death (Supplemental Figure 8). These results indicate that GFI1 was able to protect detached endothelial cells and immortalized and malignant epithelial cells from apoptosis.

At the molecular level, GSEA of the transcriptional profile of control or *GFI1*-expressing A549 cells demonstrated that when forced into suspension for 48 hours, mitochondrial gene expression and mitochondrial-related pathways were significantly downregulated upon GFI1 ectopic expression (Figure 2E and Supplemental Table 3), supporting the observation that GFI1 protected detached epithelial cells from anoikis.

We then studied the ability of A549, H460, and H1155 cells to form acinus-like spheroids in 3D basement membrane gels, which requires loss of inner cell mass through anoikis (20, 21). While A549 and H460 cells developed into well-formed hollow acini, GFI1 overexpression resulted in disrupted acinus formation with luminal filling and lack of polarity (Figure 2F and Supplemental Figure 9A). These results confirmed the function of GFI1 in inducing anoikis resistance. Consistently, H1155 cells formed grape-like spheroids in 3D basement membrane gels. However, deletion of GFI1 impaired spheroid formation (Figure 2F), confirming the observation that GFI1 enhances H1155 cell proliferation. As a third test of anchorage sensing, we examined the effect of GFI1 on anchorage-independent proliferation and found that GFI1-expressing cancer cells (A549-GFI1, H460-GFI1, H1155) formed significantly more colonies in soft agar than GFI1-negative cancer cells (A549, H460, H1155-*GFI1*-KO) (Figure 2G and Supplemental Figure 9B).

### GFI1 downregulates a group of cell-adhesion related genes.

**Figure 3:**
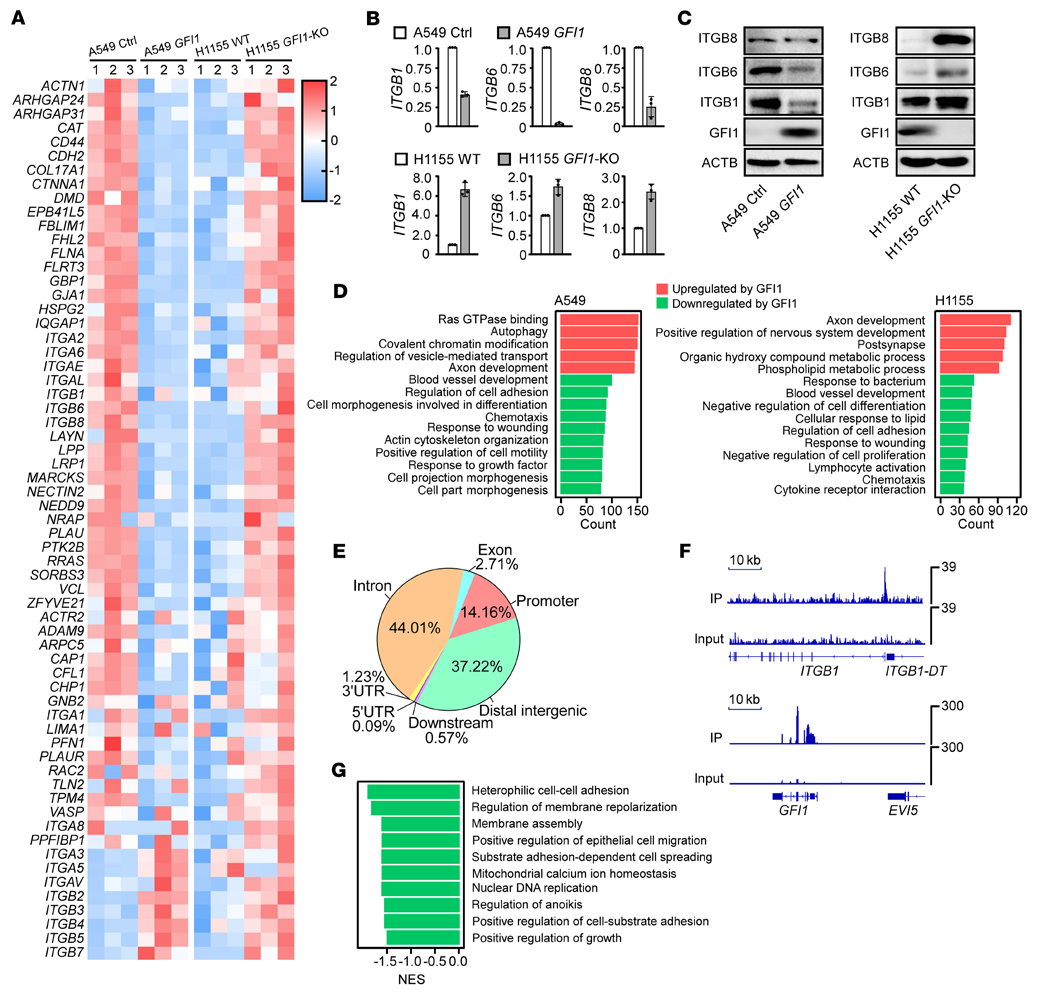
*GFI1* expression downregulates cell-adhesion genes and pathways. (**A**) The heatmap of adhesion-gene expression in A549, *GFI1*-expressing A549, H1155, and *GFI1*-KO H1155 cells. (**B**) RT-PCR showing relative transcription of the indicated genes. (**C**) Immunoblot showing expression of ITGB1, ITGB6, ITGB8, GFI1, and ACTB. The blots were generated from the same sample preparation and run at the same time. Two independent experiments were performed. See complete unedited blots in the supplemental material. (**D**) Gene ontology analysis of *GFI1*-expressing A549 cells and GFI1-deleted H1155 cells. The top 10 pathways are shown. (**E**) ChIP-Seq peaks were annotated to genomic features (promoter, 5′UTR, 3′UTR, intron, exon, distal intergenic, downstream) using the software ChIPseeker with GRCh38/hg38 as the reference genomes. (**F**) ChIP-Seq shows association of GFI1 in the indicated genes. (**G**) Gene set enrichment analysis of the GFI1-bound/-regulated genes was performed by combining ChIP-Seq and RNA-Seq. Selected downregulated pathways are shown.

To further investigate the molecular mechanism underlying the action of GFI1, we overexpressed GFI1 in A549 cells and deleted GFI1 in H1155 cells and tested the transcriptomic change by RNA-Seq. The genes downregulated in A549 cells upon GFI1 overexpression and upregulated in H1155 cells upon GFI1 deletion included cell-ECM adhesion receptors *ITGB1*, *ITGB6*, *ITGB8*, *ITGA1*, *ITGA2*, and *ITGA6* and cell-adhesion regulatory genes *FLNA* and *GBP1* (Figure 3A and Supplemental Figure 10). GFI1-induced downregulation of *ITGB1*, *ITGB6*, *and ITGB8* were confirmed at both the RNA and protein level (Figure 3, B and C). Gene ontology (GO) analysis revealed that the top 10 pathways downregulated by GFI1 in both A549 and H1155 cells included cell adhesion pathways and cell motility pathways, in keeping with the alterations in cellular morphology and adhesion. The GFI1-upregulated pathways in A549 and H1155 cells are related to axon development (Figure 3D and Supplemental Tables 4 and 5).

We next used ChIP-Seq to identify GFI1-bound genes in GFI1-FLAG–expressing A549 cells. We found 14.16% of GFI1 peaks were annotated to promoters (accounts for 12,267 genes), among which 1841 genes were upregulated and 2386 genes were downregulated upon GFI1 overexpression (Figure 3E). As expected, we observed enrichment of GFI1 in the *GFI1* locus, which is known to be autoregulated (22, 23). GFI1-bound/-downregulated genes included cell-ECM adhesion receptor genes *ITGA2*, *ITGA6*, *ITGB1*, and *ITGB8*; adhesion regulatory genes *ACTN4* and *FLNA*; cell surface adhesion genes *VCAM1* and *CEACAM6*; and DNA replication genes *MCM4*, *MCM5*, *MCM6*, *RFC4*, *BCL6*, and *DBF4* (Figure 3F). GSEA of GFI1-bound/-downregulated genes revealed downregulation of the substrate adhesion-dependent cell spreading pathway, the cell-substrate adhesion pathway, and nuclear DNA replication pathway (Figure 3G and Supplemental Table 6), again linking GFI1 to the regulation of the substrate adhesion pathways and providing molecular backgrounds of GFI1-repressed proliferation.

### GFI1 promotes lung metastasis in vivo.

**Figure 4:**
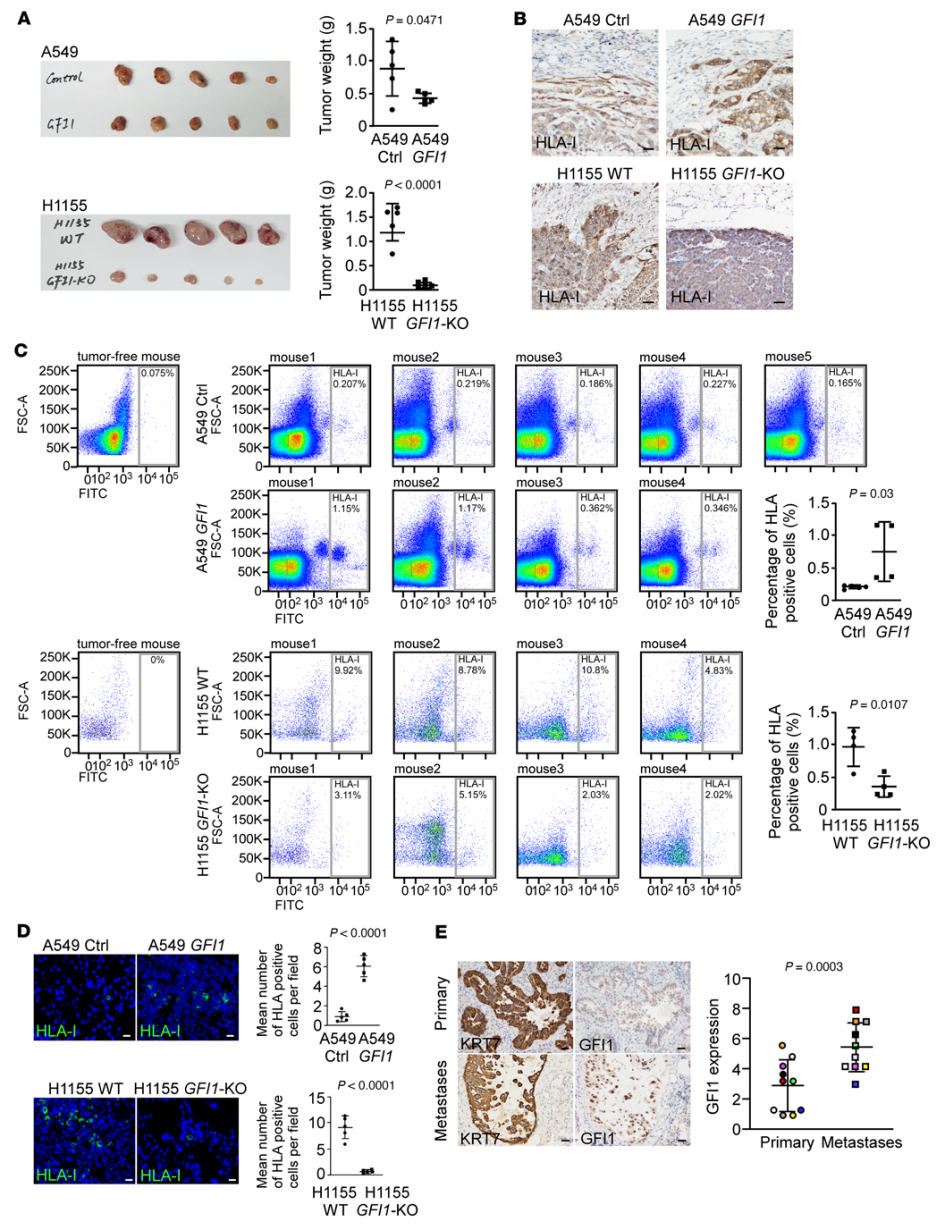
GFI1 promotes the seeding of cancer cells in the lung. (**A**) A549, *GFI1*-expressing A549, H1155, and *GFI1*-KO H1155 cells were mixed with cancer-associated fibroblasts and subcutaneously injected into 8-week-old female BALB/c nude mice. Eight weeks after the inoculation of A549 or *GFI1*-expressing A549 cells and 3 weeks after the inoculation of H1155 or *GFI1*-KO H1155 cells, the mice were euthanized and analyzed. Subcutaneous tumors are shown. (**B**) IHC staining for HLA-I was performed in the subcutaneous tumor tissue sections. GFI1 expression shifted the subcutaneous tumors from the expansive to the invasive phenotype. Scale bars: 50 μm. (**C**) Blood was collected, and cells were stained for HLA-I. The percentage of HLA-I–positive circulating tumor cells was analyzed by FACS. Tumor-free mice were used as a negative control to determine the gating. (**D**) Immunofluorescence for HLA-I was performed in lung tissue sections. The number of HLA-I–positive cells per field was counted in 10 fields from each section, and 10 sections per mouse were used. Mean values of HLA-I–positive cells/field of individual mouse were used for statistical analysis by unpaired 2-tailed Student’s *t* test. Scale bars: 20 μm. (**E**) Scores of GFI1 staining in 10 primary human NSCLCs and their matched lymphatic metastases from patients whose primary tumors expressed GFI1 were compared. Scale bars: 50 μm.

We next assessed the function of GFI1 in the regulation of cancer metastasis in vivo by utilizing a mouse xenograft model. We overexpressed GFI1 in A549 and H460 cells, mixed them with cancer-associated fibroblasts (CAFs), and subcutaneously inoculated the mixed cells into BALB/c nude mice. We also subcutaneously injected H1155*-GFI1*-KO cells mixed with CAFs into BALB/c nude mice. KO of *GFI1* significantly suppressed growth of H1155 tumors (Figure 4A). However, overexpression of *GFI1* had no effect on growth of H460 and A549 tumors (Figure 4A and Supplemental Figure 11A). These data are consistent with the in vitro proliferation data, indicating that GFI1 acts differently from proliferation in a different context.

Notably, when we stained subcutaneous tumor tissue sections for HLA-I, which can distinguish human tumor cells from mouse cells, we found that although it exerted a negative role in cell invasion in vitro, GFI1 expression caused a shift from the expansive to the invasive phenotype in all studied subcutaneous tumors (Figure 4B and Supplemental Figure 11B). Consistent with this phenotype, when we stained cells collected from the angular vein of the mice for HLA-I and tested circulating tumor cells using FACS, we found that mice bearing *GFI1*-expressing cancer cells contained significantly more circulating tumor cells than those bearing cancer cells without GFI1 (Figure 4C). Additionally, we also detected more infiltrated HLA-I–positive human cancer cells by immunostaining in the lung tissue sections in mice bearing *GFI1*-expressing cancer cells than in those bearing GFI1-negative cancer cells (Figure 4D and Supplemental Figure 11C). Thus, GFI1 enhanced cell invasiveness and the ability to infiltrate the lung tissue. In agreement with the results from the xenograft assay, in a different animal model whereby H1155 and H1155-*GFI1*-KO cells were intravenously injected into BALB/c nude mice, heavy lung metastasis was detected in mice injected with H1155 cells but not in mice injected with H1155-*GFI1*-KO cells at 7 weeks after injection (Supplemental Figure 12). In addition, immunostaining of GFI1 in tissue sections of 18 primary human NSCLCs and their matched lymphatic metastases showed that among the 10 patients whose primary tumors expressed GFI1, 8 had more GFI1-expressing lung cancer cells in lymphatic metastases than their corresponding primary tumors (Figure 4E).

### GFI1 promotes anchorage independence by activating ERK.

**Figure 5:**
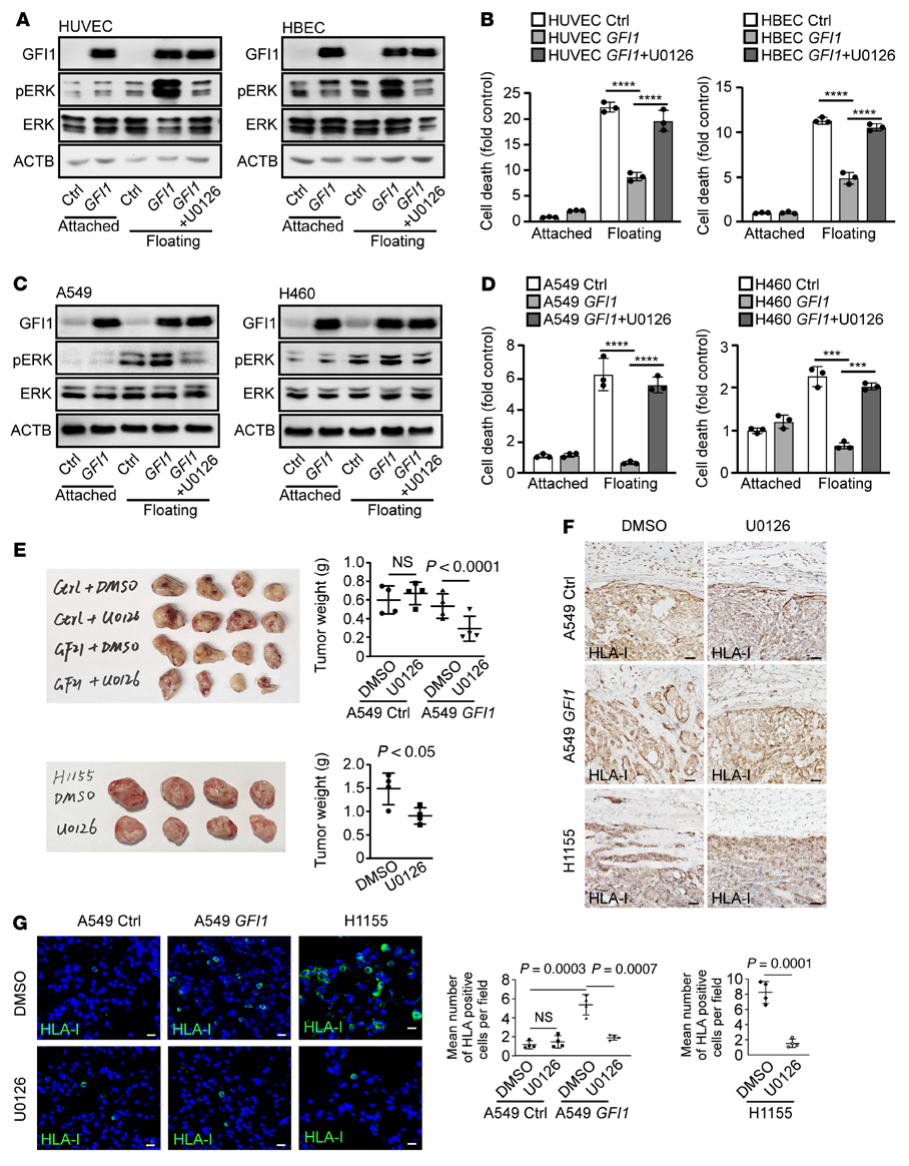
GFI1 confers anchorage independence by activating the ERK pathway. (**A**) Cells were overexpressed with GFI1 and treated with 10 μM U0126. Protein levels of GFI1, pERK, ERK, and ACTB were assessed 16 hours after the cells were under attached or floating conditions. The blots were generated from the same sample preparation and run at the same time. Two independent experiments were performed. See complete unedited blots in the supplemental material. (**C**) Cells were overexpressed with GFI1 and treated with 10 μM U0126. Protein levels of GFI1, pERK, ERK, and ACTB were assessed 24 hours after under attached or floating conditions. The blots were generated from the same sample preparation and run at the same time. Two independent experiments were performed. (**B** and **D**) Cell death of indicated cells under conditions shown as in **A** and **C**. Mean ± SD represents 3 replicates in 1 experiment. Three independent experiments were performed. Data shown as mean ± SEM. ***P < 0.001; ****P < 0.0001 (1-way ANOVA test with post hoc contrasts by Tukey’s test). (**E**) 8-week-old female BALB/c nude mice were subcutaneously inoculated with A549, *GFI1*-expressing A549, or H1155 cells mixed with cancer-associated fibroblasts and treated with ERK inhibitor U0126. Six weeks later, the mice were euthanized and analyzed. Subcutaneous tumors are shown. (**F**) Representative images of IHC staining of HLA-I in subcutaneous tumor tissue sections. Scale bars: 50 μm. (**G**) Immunofluorescence for HLA-I was performed in lung tissue sections. The number of HLA-I–positive cells per field was counted in 10 fields from each section, and 10 sections per mouse were used. Scale bars: 20 μm. Results expressed as mean number of HLA-I–positive cells/field of individual mouse. Multiple comparisons were performed with a 1-way ANOVA test with post hoc contrasts by Tukey’s test. Unpaired 2-tailed Student’s *t* test was used to compare the means of 2 populations.

Interactions of integrins with ECM activate the PI3K/AKT signaling and the classical ERK pathway to provide survival signals (24–26). FAK can also be activated by integrins in response to adhesion and in turn interacts with the PI3K/AKT and ERK signaling pathways (5, 27, 28). Aside from these outside-in survival signaling pathways, p66^Shc^, an adapter protein that localizes to focal adhesions, can sense the change of mechanical stress and induce anoikis upon detachment (29). We found that GFI1 overexpression failed to alter the expression of p66^Shc^ or the level of AKT phosphorylation (Supplemental Figure 13, A and B). Instead, it significantly elevated both FAK and ERK phosphorylation (Figure 5A and Supplemental Figure 13C). Consistent with the increase in ERK phosphorylation, the treatment of cells with U0126, a specific ERK signaling inhibitor, restored anoikis in *GFI1*-expressing cancer cells (Figure 5, A–D). However, inhibitors of FAK signaling (FAK inhibitor 14, defactinib, dasatinib) exerted little effect (Supplemental Figure 13, C–F). These results indicate that GFI1 reduced the sensitivity of cells to anoikis by activating the ERK pathway.

To further determine whether GFI1-caused in vivo phenotypes result from GFI1-induced ERK activation, we used U0126 in the mouse xenograft model. One week after subcutaneous inoculation of A549 and GFI1-expressing A549 and H1155 cells, we administered ERK inhibitor U0126 to mice intraperitoneally at 25 μmol/kg twice a week until the end of this assay. Interestingly, while U0126 had no effect on the tumor growth, invasion, and lung infiltration for A549 tumors, it significantly suppressed all these phenotypes in GFI1-expressing A549 tumors and H1155 tumors (Figure 5, E–G), indicating that GFI1-caused tumor invasion and metastasis resulted from GFI1-induced ERK activation. Thus, GFI1 promotes tumor progression through enhancing ERK pathway, and this leads to an ERK signaling addiction in tumor cells.

### GFI1 activates the ERK pathway through RASGRP2 upregulation.

**Figure 6:**
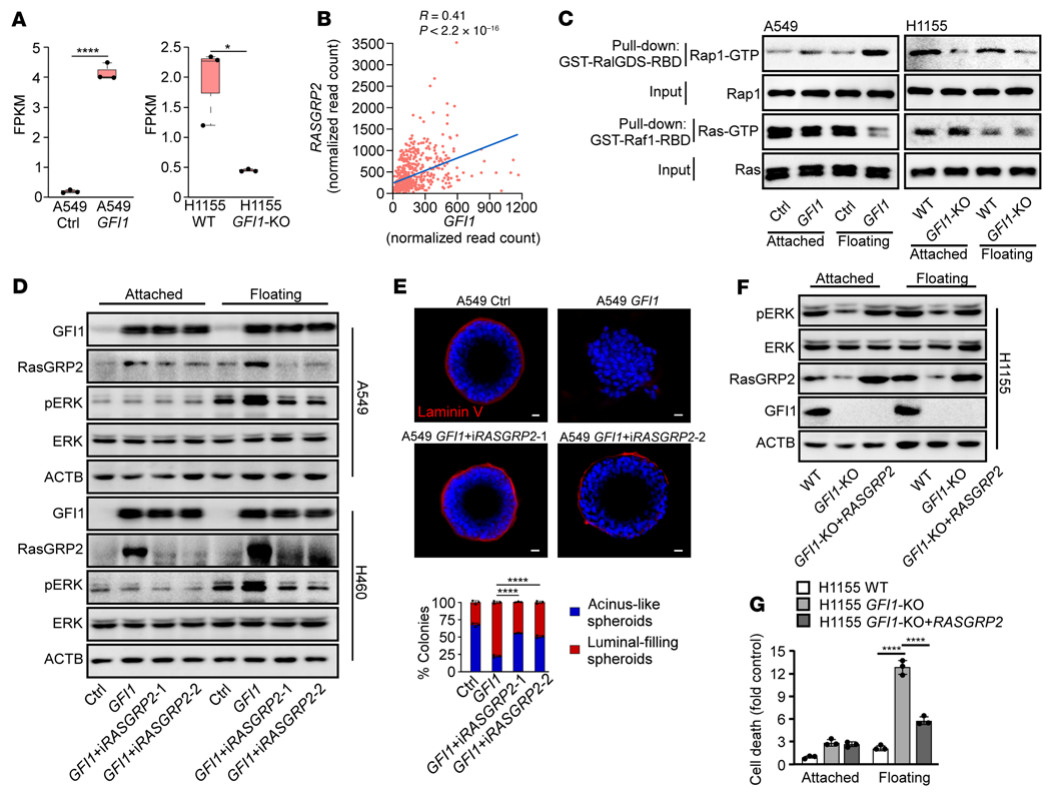
GFI1 activates the ERK pathway by upregulating RasGRP2. (**A**) Expression level of *RASGRP2* in RNA-Seq. *P < 0.05; ****P < 0.0001 (unpaired 2-tailed Student’s *t* test). (**B**) Gene expression correlation between *GFI1* and *RASGRP2* in patients with lung adenocarcinoma (LUAD) from the TCGA database. (**C**) Immunoblot showing Rap1-GTP pulled down by GST-RalGDS-RBD and Ras-GTP pulled down by GST-Raf1-RBD in the indicated cells. (**D**) Indicated cells were subjected to immunoblot analysis of pERK level. (**E**) Indicated cells were cultured in Matrigel for 8 days. Confocal midpoint slices of DAPI- and laminin V–stained acinus are shown. Scale bars: 20 μm. Mean ± SD represents 10 visualized areas in 1 experiment. Three independent experiments were performed. ****P < 0.0001 (1-way ANOVA test with post hoc contrasts by Tukey’s test). (**F**) Immunoblot showing expression of pERK, ERK, RasGRP2, GFI1, and ACTB in the indicated cells. See complete unedited blots in the supplemental material. (**G**) The cell death of indicated cells was assessed after 24 hours. Mean ± SD represents 3 replicates in 1 experiment. Three independent experiments were performed. ****P < 0.0001 (1-way ANOVA test with post hoc contrasts by Tukey’s test).

To understand the underlying mechanism by which GFI1 activates the ERK pathway, we analyzed our RNA-Seq data and found that *RASGRP2* was upregulated by 20-fold in A549 cells after lentiviral transduction of GFI1 but decreased by 4-fold in H1155 cells with GFI1 deletion (Figure 6A). Analysis of The Cancer Genome Atlas also revealed the positive correlation between *GFI1* and *RASGRP2* in lung adenocarcinomas (Figure 6B). In addition, ChIP-qPCR revealed that GFI1 expression increased association of H3K27ac and H3K4me3 and decreased association of H3K27me3 with the promoter and enhancer region (Supplemental Figure 14), confirming the role of GFI1 in *RASGRP2* upregulation. 

RasGRP2 is a Ras GDP-releasing factor (GRP) generally used by hematopoietic cells and neurons to activate Rap1, a small GTPase known to activate B-raf, resulting in the activation of MAP2K (MEK) and the ERK signaling pathway (30). Activation of RasGRP2 requires PKA (31). PKA, which can be activated in detached epithelial cancer cells, phosphorylates RasGRP2 at S116, S117, S554, and S586, which subsequently activates RAP1 (32). Indeed, overexpression of GFI1 increased Rap1 activation but not Ras activation in suspended A549 cells (Figure 6C). In contrast, KO of GFI1 decreased Rap1 activation in H1155 cells (Figure 6C). To further assess whether GFI1 activates the ERK pathway via RasGRP2, we lentivirally transduced *RASGRP2* shRNA into GFI1-expressing A549 and H460 cells and *RASGRP2-*expressing cassette into H1155-*GFI1*-KO cells and determined the activity of ERK and the resulting anoikis sensitivity. RasGRP2 knockdown nearly completely blocked GFI1-induced ERK activation in suspended cancer cells and restored anoikis of A549 and H460 cells and the ability of A549 cells to form organized acini in the basement membrane (Figure 6, D and E). Consistently, deletion of GFI1 in H1155 cells repressed ERK activity. Re-expression of RasGRP2 restored ERK activation and anoikis resistance (Figure 6, F and G). Thus, GFI1 activated the ERK pathway through RasGRP2 upregulation and consequently conferred cancer cells with anchorage independence.

A549 cells harbor Ras mutation. We next explored whether A549 cells depend on RasGRP2 to activate ERK without GFI1. Knockdown of RasGRP2 reduced ERK activity in suspended A549 cells and increased cell death in both attached and suspended A549 cells (Supplemental Figure 15), suggesting that RasGRP2 also contributes to ERK activation in suspended A549 cells without GFI1 and that RasGRP2 may promote cell survival through an ERK-independent pathway in attached A549 cells.

### GFI1 enhances RASGRP2 transcription by mediating long-range enhancer-promoter interaction.

**Figure 7:**
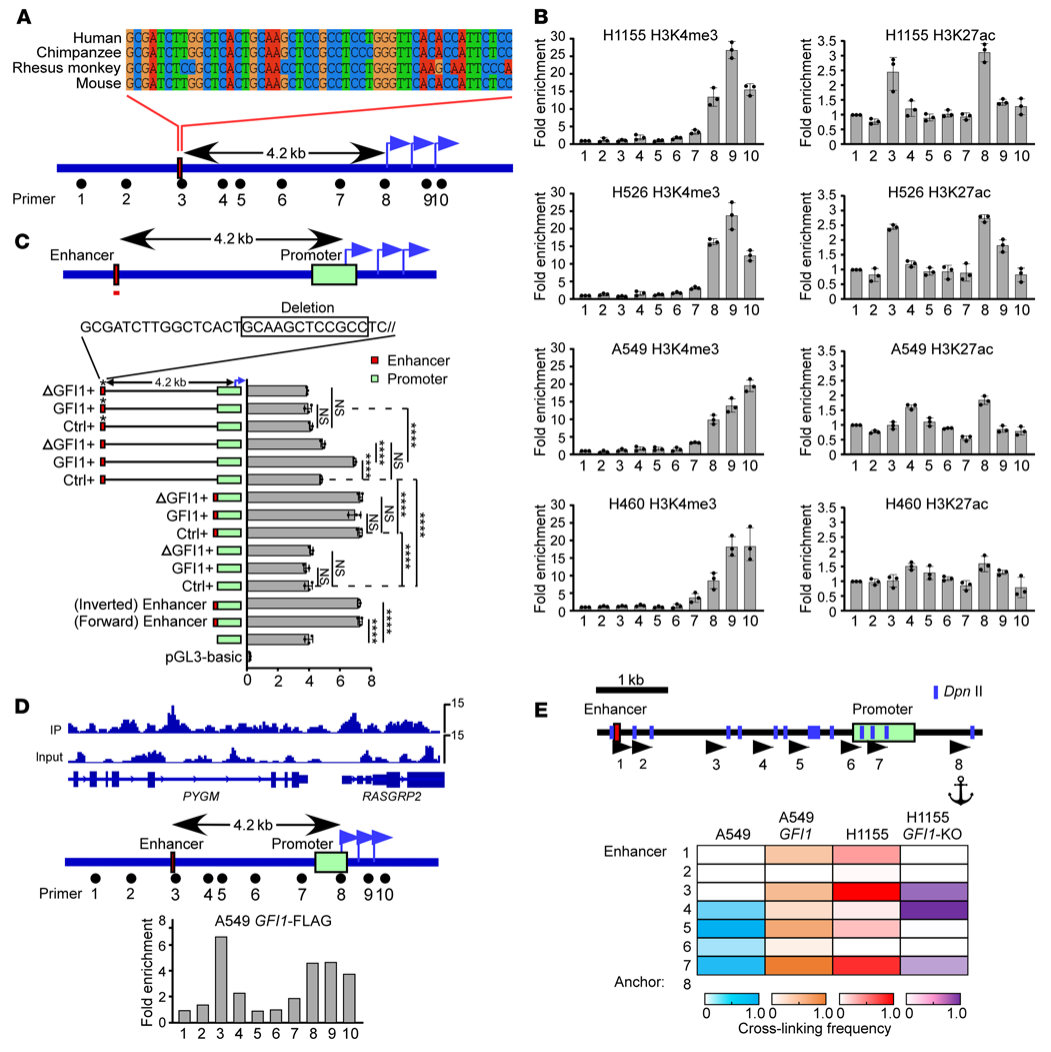
GFI1 establishes the interaction between the enhancer and promoter of *RASGRP2*. (**A**) Alignment of human, chimpanzee, Rhesus monkey, and mouse *RASGRP2* genes revealed a 50 bp conserved sequence residing within the noncoding region 4 kb upstream from the *RASGRP2* transcriptional start site. (**B**) ChIP shows distribution of H3K4me3 and H3K27ac histone modifications in H1155, H526, A549, and H460 cells. The location of primers is shown in **A**. (**C**) Bar graphs show luciferase reporter activity with ectopic placement of the 50 bp conserved DNA sequence (red line in upper diagram) adjacent to the promoter, ectopic placement of mutant GFI1 lacking DNA-binding activity, or mutation of GFI1 binding motif within the enhancer region. Luciferase activity was normalized to Renilla signals. Mean ± SD represents 3 replicates in 1 experiment. Three independent experiments were performed. ****P < 0.0001 (1-way ANOVA test with post hoc contrasts by Tukey’s test). (**D**) ChIP-Seq showing enrichment of GFI1 in the *RASGRP2* locus (top). ChIP-QPCR analysis showing the association of GFI1-Flag in transfected A549 cells with regions 3 and regions 8–10 at the (bottom). The location of primers is shown (middle). (**E**) 3C was used to calculate the cross-linking frequency between chromatin segments to assess the proximity in A549, H1155, *GFI1*-expressing A549, and *GFI1*-deleted H1155 cells. Vertical lines represent *Dpn* II restriction sites; arrows indicate PCR primer sites and direction. Anchor symbols mark the anchoring primer. Heatmaps showing the cross-linking frequency between the *RASGRP2* promoter and other *Dpn* II–defined segments.

We then explored the mechanism by which GFI1 upregulates *RASGRP2* expression. Alignment of human, chimpanzee, rhesus monkey, and mouse *RASGRP2* genes revealed a 50 bp conserved sequence residing within the noncoding region 4.2 kb upstream from the *RASGRP2* transcriptional start site (TSS), marking one potential cis-regulatory element (Figure 7A, site 3). We next used ChIP to scan the *RASGRP2* gene for H3K27ac, a modified histone that is frequently associated with human active enhancers and active promoters, and H3K4me3, a modified histone that marks active or poised promoters, to determine the function of this putative cis-regulatory element. We found that site 8 was co-occupied by H3K27ac and H3K4me3 in all cells we studied, indicating that among 3 *RASGRP2* promoters, TSS1 was active in lung cancer cells and that although *RASGRP2* transcription activity was lower in A549 and H460 cells than in H1155 and H526 cells, its promoter was still in an active state. In contrast, site 3 was enriched with H3K27ac but not H3K4me3 in H1155 and H526 cells only but not in A549 and H460 cells, suggesting that this putative cis-regulatory element was an enhancer and that this enhancer was inactive in A549 and H460 cells (Figure 7B). Indeed, this 50 bp DNA sequence, when placed adjacently upstream of the *RASGRP2* promoter either in forward or reverse orientations, was able to increase the promoter activity in a luciferase reporter assay (Figure 7C). Interestingly, cotransfection of GFI1 had no effect on *RASGRP2* promoter activity if the luciferase reporters contained the *RASGRP2* promoter only or the *RASGRP2* promoter with the enhancer adjacently placed upstream of the promoter. However, cotransfection of GFI1 was able to increase *RASGRP2* promoter activity if the enhancer was in its original position (4.2 kb upstream from the *RASGRP2* promoter), and this effect required binding of GFI1 to the enhancer region because cotransfection of GFI1 mutant (lacking DNA-binding activity) or deletion of GFI1 binding motif within the enhancer region blocked elevation of *RASGRP2* promoter activity (Figure 7C). ChIP-Seq showed the occupancy of transfected GFI1 in the *RASGRP2* promoter and enhancer region in A549 cells, which was confirmed by ChIP-qPCR (Figure 7D). Chromosome conformation capture (3C) assay revealed the enhancer-promoter colocalization in GFI1-expressing cells (H1155, A549-GFI1) but not in GFI1-negative cells (A549) (Figure 7E). These collective data suggest that GFI1 may bind to the enhancer and mediate the enhancer-promoter physical interaction to upregulate *RASGRP2* expression.

## Discussion

Metastasis is the leading cause of cancer-related death. For metastasis to succeed, cancer cells must reduce their substrate adhesion and gain resistance to anoikis to dissociate from their primary site and survive during circulation in the vascular system (4, 33). This phenotypic change shares many similarities with early hematopoiesis. In this study, we demonstrated that these two biological processes used a common transcription factor, GFI1, to gain anchorage independence.

GFI1 is a key regulator of early hematopoiesis (34). Upon GFI1 expression, endothelial cells downregulate the expression of endothelial genes and undergo morphological changes from adherent endothelium cells to round hematopoietic cells. Hemogenic endothelium in *GFI1*-KO mice can differentiate into hematopoietic progenitor cells but these hematopoietic progenitor cells maintain the adherent phenotype of endothelial cells and fail to disseminate from their developmental niche. By contrast, in the absence of RUNX1, a transcription factor that activates GFI1 expression and drives hematopoiesis, enforced GFI1 expression triggers the loss of endothelial phenotype and the formation of round cells. However, these round cells fail to generate hematopoietic colonies (34). In addition, introduction of *GFI1* together with *RUNX1*, *FOSB,* and *SPI1* successfully converts adult endothelial cells to hematopoietic stem cells (35). These findings suggest that GFI1 functions as a decisive switch in the transition from anchorage dependence to anchorage independence during hematopoiesis.

Recent reports have shown that GFI1 acts as an oncoprotein in multiple malignancies, such as leukemia and several solid tumors, by promoting cell proliferation or suppressing the immune system (36–39). Consistently, we showed that GFI1 also functions as an oncoprotein in lung cancer. GFI1 expression is particularly high in SCLCs, consistent with previous findings that GFI1 expression correlates with the neuroendocrine phenotype of human cancer (40). The function of GFI1 in lung cancer cells is to promote dissemination of cancer cells from their primary site, similar to its physiological function of promoting dissemination of hematopoietic progenitor cells from their developmental niche during hematopoiesis.

We showed that GFI1 represses expression of multiple cell adhesion–related genes, including integrins whose interactions with ECM proteins play an important role in cell survival. Normally, integrin-mediated cell-ECM contacts activate FAK, Src, ILK, and subsequently Akt and Erk to protect cells from apoptosis (41). However, GFI1-induced substrate detachment escapes anoikis because GFI1 concomitantly upregulates RasGRP2, which in turn activates Rap1 and its downstream ERK signaling pathway to ensure survival of detached cancer cells. Interestingly, RasGRP2 is normally expressed in the vascular system and its genetic variation is associated with platelet dysfunction and bleeding (42, 43).

The ERK pathway is active in the majority of human lung cancers, especially in lung adenocarcinomas, because its upstream cues Ras and EGFR are frequently mutated (44, 45). Different from this oncomutation-activated Ras/ERK pathway, GFI1-caused ERK activation in detached cells is mediated through RasGRP2 and Rap1. Although Ras and Rap1 have sequence similarities and Rap1 can compete with Ras for Raf1, their activators and effectors are generally distinct (46). It is noteworthy that despite bearing Ras mutation, A549 tumors were not responsive to an ERK signaling inhibitor. However, GFI1-expressing tumors, including A549-GFI1 and H1155 tumors, were responsive to an ERK signaling inhibitor. Additionally, although A549 cells bear Ras mutation, we found that overexpression of GFI1 can further increase ERK activation. These data indicate that GFI1-induced activation of RasGRP2/Rap1 resulted in higher ERK activation and ERK signaling dependency in tumor cells. This is presumably due to the different role of Ras and Rap1 in ERK activation: Ras initiates whereas Rap1 sustains ERK signaling (47). Activated ERK directs the phosphorylation of the RasGEF Sos to terminate Ras-dependent ERK activation (48).

GFI1 contains the “SNAG” transcriptional repressor domain in its N-terminus, which functions mostly as a transcriptional repressor. GFI1 binds to DNA; recruits chromatin-modifying enzymes, such as ETO (49), histone deacetylase (HDAC) (50), G9A histone lysine methyltransferase (50), lysine-specific demethylase-1 LSD1 (51), and Ajuba (52); and represses gene expression. In our study, we showed that GFI1 inhibited a number of genes, including adhesion-related genes and apoptotic genes when expressed in lung cancer cells. However, we also found that GFI1 can upregulate *RASGRP2* transcription. It has been reported that GFI1 activates gene transcription, for example, the ST2 gene in group 2 innate lymphoid cells (ILC2) and the secondary granule protein (SGP) gene in neutrophil cells (53, 54). However, the underlying mechanism by which GFI1 activates gene transcription is not clear. Our studies showed that GFI1 bound to an upstream enhancer and promoter of *RASGRP2*, leading to their colocalization. This suggested a model whereby GFI1 may upregulate *RASGRP2* by reconfiguring its gene structure. How GFI1 reconfigures *RASGRP2* gene structure and upregulates its expression requires further experimentation. It is possible that GFI1 functions as a transcriptional activator and directly activates *RASGRP2*. It is also possible that GFI1 upregulates *RASGRP2* through repressing transcriptional inhibitors that mediate the activation of *RASGRP2*.

In summary, we have unveiled a potentially novel GFI1-dependent mechanism by which epithelial cancer cells gain anchorage independence. GFI1 serves as a key regulatory molecule of anchorage independence and metastasis and exerts its function by triggering the RasGRP2/RAP1/ERK signaling cascade. We therefore unveiled a molecular signaling network that regulates GFI1 induction of anoikis resistance and thereby provide a potentially new therapeutic strategy for patients with GFI1-positive lung cancer.

## Methods

### Reagents and tools.

Reagents and tools are listed in Supplemental Table 7.

### Human samples.

Human lung cancer tissues were collected at Tianjin Medical University Cancer Hospital. The use of all human lung cancer specimens was approved by the IRB of Tianjin Medical University Cancer Hospital. Informed consent was obtained from all patients, and samples were deidentified prior to analysis.

### Cell lines and animals.

HBECs are normal human bronchial epithelium immortalized by hTERT and CDK4 and were obtained from Jerry Shay (University of Texas Southwestern Medical Center, Dallas, Texas) in 2008. HUVECs were purified from umbilical tissues as described in a previous report (55) and maintained in EGM-2 Bullet kit (CC3162, Lonza). A549 (RRID: CVCL_0023), H460 (RRID: CVCL_0459), H1299 (RRID: CVCL_0060), H1155 (RRID: CVCL_1456), H69 (RRID: CVCL_1579), H526 (RRID: CVCL_1569), H82 (RRID: CVCL_1591), MCF7 (RRID: CVCL_0031), MDA-MB-231 (RRID: CVCL_0062), SKOV3 (RRID: CVCL_0532), HT-29 (RRID: CVCL_0320), OVCA432 (RRID: CVCL_3769), HepG2 (RRID: CVCL_0027), Hep3B (RRID: CVCL_0326), and HCT8 (RRID: CVCL_2478) cells were obtained from ATCC within the past 10 years and maintained in ATCC-recommended media supplemented with 10% FBS.

Eight-week-old female BALB/c nude mice were obtained from Beijing Vital River Laboratory Animal Technology Co., Ltd.

### Transcription factor–binding motif enrichment.

Analysis of transcription factor–binding motifs in transcriptional profiles of 29 human SCLC lines compared with 118 human NSCLC lines was performed using GSEA software (v4.1.0) with default parameters. Gene sets C3 (TFT: transcription factor targets) from MSigDB (https://www.gsea-msigdb.org/gsea/msigdb/genesets.jsp?collection=TFT) were included in the analysis.

### Immunofluorescence and IHC analysis.

Lung cancer tissues were obtained from Tianjin Medical University Cancer Institute and Hospital. Samples were fixed in 4% paraformaldehyde at 4°C overnight, dehydrated in gradient alcohol and xylene, and embedded in paraffin. Paraffin blocks were cut into 5 μm sections.

### Tissue microarrays.

The breast cancer tissue microarray (HBreD140Su07) and ovarian cancer tissue microarray (HOvaC151Su01) were purchased from Shanghai Xinchao Biological Technology Co., Ltd. Antigen retrieval was performed in 0.1 M citrate buffer (pH 6.0) by microwaving. Slides were blocked for 1 hour at room temperature using 10% normal goat serum in 0.1 M PBS containing 0.1% Triton X-100. For IHC staining, endogenous peroxidase activity was blocked in 3% hydrogen peroxide. Then, tissue sections were immunostained with proper primary antibodies at 4°C overnight and with appropriate HRP-conjugated secondary antibodies (1:500) in blocking buffer for 2 hours at room temperature and analyzed by DAB staining. For immunofluorescence staining, after immunostaining with primary antibodies, appropriate secondary antibodies conjugated to Alexa Fluor 488 or Alexa Fluor 555 were used at a dilution of 1:500 in blocking buffer. Nuclei were counterstained with DAPI (1 μg/mL). The fluorescence images were observed under a confocal microscope (Zeiss, LSM800). Primary antibodies are listed in Supplemental Table 7. Summaries of the lung tumor specimen data, breast tumor specimen data, and ovarian tumor specimen data are shown in Supplemental Tables 8–10, respectively.

Quantitative scoring of tissue sections was performed in a blinded fashion. The staining intensity and proportion of the nuclear GFI1 were scored separately and contributed to the aggregate score. Median value was used as the cutoff to analyze the correlation between GFI1 expression and survival of cancer patients. Kaplan-Meier survival curves were analyzed using Mantel-Cox log-rank tests, and hazard ratios were calculated using Mantel-Haenszel tests (GraphPad Prism 9.0.0).

### Overexpression of GFI1.

Human *GFI1* was amplified from HUVEC cell cDNA. PCR fragments of *GFI1* were ligated into the lentiviral shuttle pCCL.PPT.hPGK.IRE-S.eGFP/pre containing GFP and were used to produce lentivirus in Phoenix-293 cells with the packaging plasmids pMD2.BSBG, pMDLg/pRRE, and pRSV-REV. Lentivirus-infected cancer cells were directly used in the proliferation assay, wound healing assay, Matrigel invasion assay, soft agar assay, and 3D culture. FACS-sorted GFP^+^ cells were used for RNA-Seq and animal assays.

### CRISPR/Cas9-mediated GFI1 promoter deletion.

sgRNAs specific to the *GFI1* promoter region were designed from http://crispr.mit.edu, and 2 pairs of sgRNAs with minimized off-target effects were selected and are shown in Supplemental Table 11. DNA oligonucleotides were synthesized and ligated into *Bbs I*–digested pSpCas9(BB)-2A-GFP (PX458) after annealing. The *GFI1* gene was destroyed in H1155s by the means of CRISPR/Cas9 upon dual transfection of 2 plasmids containing the Cas 9 gene and 2 different sgRNAs specific to 2 regions of the *GFI1* promoter. Monoclonal GFP^+^ clones were sorted by FACS.

### Adhesion assay.

The concentrations of fibronectin, collagen I, and laminin were 10 μg/mL, 2 μg/mL, and 10 μg/mL, respectively. They were added to a 6-well plate and incubated at 4°C overnight. For blocking, the 6-well plates were incubated with 0.2% sterile BSA for 2 hours. Cells were detached with 0.25% trypsin-EDTA, centrifuged (300*g* for 3 min), resuspended, and counted. Single cells (1 × 10^5^ per well in triplicate) mixed into 0.5 mL complete medium were incubated in coated 6-well plates for 30 minutes. The adherent cells were stained with 0.1% crystal violet, photographed, and counted.

### Determination of anoikis.

A total of 2 × 10^5^ cells in each group were cultured on ultra-low attachment or cell culture–treated 24-well plates. Next, 24 hours after the culture, anoikis was assessed using Cell Death Detection ELISA PLUS kit following the manufacturer’s instructions.

### 3D Matrigel culture.

Detached cells (2 × 10^3^ per well in triplicate) were mixed into 0.4 mL of RPMI 1640 or DMEM medium supplemented with 5% chilled growth factor–reduced Matrigel and 2% FBS and cultured in 24-well ultra-low attachment plates (Corning) at 37°C for 8 days. Experiments were performed in triplicate. The spheroids were collected, centrifuged (300*g* for 1 min), and dropped on the slide. The spheroids on the slide were air-dried, fixed with 4% paraformaldehyde, immunostained with antibodies against laminin V and ITGB1, and mounted with Fluoroshield with DAPI. Colonies of more than 50 μm were counted.

### Soft agar.

Cells (10^4^ per well in triplicate) were resuspended in RPMI 1640 or DMEM containing 10% FBS with 0.7% agarose and layered on top of 1.2% agarose in RPMI 1640 or DMEM on 6-well plates. Cells were cultured for 2 weeks at 37°C with 5% CO_2_. Experiments were performed in triplicate. Colonies were stained, analyzed morphologically, and counted using light microscopy.

### 3D dense gel coculture.

An invasion assay was performed with a final collagen I concentration of 4 mg/mL and a final Matrigel concentration of 2 mg/mL. Neutralized mixture gel (600 μL/well) was then aliquoted onto a 0.4 μm polyester membrane. The mixed collagen-Matrigel gel was allowed to polymerize at 37°C for about 1 hour. Then, 5 × 10^5^ cells were plated on the top of gels. The gel was fed from underneath with complete medium supplemented with 1% insulin-transferrin-selenium and 2 mM GlutaMAXTM-I. Medium was changed daily. After 8 to 10 days, gel was fixed using 4% paraformaldehyde and embedded in paraffin; 5 μm paraffin sections were subjected to H&E staining.

### BrdU proliferation assay.

A total of 10^4^ cells in each group were cultured on 96-well plates for 12 hours. Then, the cells were incubated with BrdU for 6 hours. Proliferation was assessed using a Cell Proliferation ELISA kit following the manufacturer’s instructions.

### Cell viability assay.

Single-cell suspensions were counted and resuspended in PBS at a concentration of 10^7^ cells/mL. One hundred microliters of each sample were stained with Zombie NIR Fixable Viability kit (BioLegend) following the manufacturer’s instructions. All ﬂow cytometry analyses were performed using a Fortessa ﬂow cytometer (BD Biosciences) and analyzed using FlowJo software (TreeStar).

### In vivo metastasis assay.

First, 10^6^ A549 cells expressing empty vector or *GFI1* mixed with 5 × 10^5^ human lung CAFs in 120 μL PBS containing Matrigel (1:1 vol/vol; BD Biosciences) were injected into the flank of 8-week-old female BALB/c nude mice (5 mice for control A549 cells, 5 mice for *GFI1*-expressing A549 cells). Eight weeks after the inoculation of cells, we collected peripheral blood from the angular vein of the mice. PBMCs containing circulating tumor cells were isolated by density centrifugation with Ficoll-Paque. Cells were stained with antibodies against HLA-I to distinguish human tumor cells from mouse blood cells and analyzed by FACS (we failed in collecting blood from 1 mouse carrying *GFI1*-expressing A549 cells). PBMCs from a tumor-free mouse were used as the negative control. For each sample, negative controls were used to determine the gating. The mice were euthanized. Subcutaneous tumors and lung tissues were dissected, fixed in 4% paraformaldehyde, and embedded in paraffin. Paraffin blocks were cut into 5 μm sections. Subcutaneous tumor and lung tissue sections were stained with antibodies against HLA-I.

First, 5 × 10^5^ H1155 cells or H1155*-GFI1*-KO cells mixed with 2.5 × 10^5^ human lung CAFs in 120 μL PBS containing Matrigel (1:1 vol/vol; BD Biosciences) were injected into the flank of 8-week-old female BALB/c nude mice (5 mice for H1155 cells, 5 mice for H1155*-GFI1*-KO cells). Three weeks after the inoculation of cells, we collected peripheral blood from the angular vein of the mice and analyzed circulating tumor cells as described above (we failed in collecting blood from each mouse carrying H1155 cells and H1155*-GFI1*-KO cells). The mice were euthanized. Subcutaneous tumors and lung tissues were dissected, fixed in 4% paraformaldehyde, and embedded in paraffin. Paraffin blocks were cut into 5 μm sections. Subcutaneous tumor and lung tissue sections were stained with antibodies against HLA-I.

Next, 3 × 10^5^ H460 cells expressing empty vector or *GFI1* mixed with 1.5 × 10^5^ human lung CAFs in 120 μL PBS containing Matrigel (1:1 vol/vol; BD Biosciences) were injected into the flank of 8-week-old female BALB/c nude mice (4 mice for control H460 cells, 4 mice for *GFI1*-expressing H460 cells). Three weeks after the inoculation of cells, the mice were euthanized. Subcutaneous tumors and lung tissues were dissected, fixed in 4% paraformaldehyde, and embedded in paraffin. Paraffin blocks were cut into 5 μm sections. Subcutaneous tumor and lung tissue sections were stained with antibodies against HLA-I.

Next, 10^6^ H1155 and H1155-*GFI1*-KO cells in 100 μL PBS were intravenously injected into 8-week-old female BALB/c nude mice. Seven weeks after injection, the mice were euthanized. Lung tissues were dissected, fixed in 4% paraformaldehyde, and embedded in paraffin. Paraffin blocks were cut into 5 μm sections. Lung tissue sections were subjected to H&E staining.

### U0126 treatment.

Twenty-four 8-week-old female BALB/c nude mice were randomized in each treatment group before the initiation of dosing (4 mice for each group). Next, 10^6^ A549 cells expressing empty vector or *GFI1* mixed with 5 × 10^5^ human lung CAFs in 120 μL PBS containing Matrigel (1:1 vol/vol; BD Biosciences) were injected into the flank of mice. Also, 3 × 10^5^ H1155 cells mixed with 1.5 × 10^5^ human lung CAFs in 120 μL PBS containing Matrigel (1:1 vol/vol; BD Biosciences) were injected into the flank of mice. One week after subcutaneous inoculation of tumor cells, we administered ERK inhibitor U0126 to mice. Mice were treated with vehicle (DMSO) or U0126 (25 μmol/kg) twice weekly via intraperitoneal injection. The treatment was continued until the end of the assay.

Six weeks after the inoculation of A549 cells and 3 weeks after the inoculation of H1155 cells, the mice were euthanized. Subcutaneous tumors and lung tissues were dissected, fixed in 4% paraformaldehyde, and embedded in paraffin. Paraffin blocks were cut into 5 μm sections. Subcutaneous tumor and lung tissue sections were stained with antibodies against HLA-I.

All animal procedures were approved by the IACUC at Tianjin Medical University and conformed to the legal mandates and national guidelines for the care and maintenance of laboratory animals.

### ChIP-Seq and ChIP.

*GFI1*-FLAG–expressing A549 cells were cross-linked with 1% formaldehyde for 10 minutes at room temperature. Cross-linking was terminated by 0.125 M glycine for 5 minutes. Cells were subsequently washed twice with ice-cold PBS, collected in 15 mL tubes, and pelleted by centrifugation at 800*g* for 10 minutes at 4°C. Pellets were resuspended in 750 μL of sonication buffer (100 mM NaCl, 0.1% SDS, 5 mM EDTA pH 8.0, 100 mM Tris-HCl pH 8.0, 1% Triton X-100) and sonicated to obtain fragments (100–500 bp) with SONICS ultrasonic processor. Immunoprecipitation was performed with ANTI-FLAG M2 Affinity gel, overnight at 4°C with rotation. After elution and reversal cross-linking, DNA was purified and analyzed by next generation sequencing. Quality control was performed using FastQC v0.11.8. Clean reads were mapped to the reference genome GRCh38/hg38 using Bowtie2.3.5 (56). For each sample, ChIP peaks were detected using MACS2 (57). Bigwig files were generated by deepTools. ChIP-Seq tracks were visualized in IGVtools. Peaks were annotated to genomic features (promoter, 5′UTR, 3′UTR, intron, exon, distal intergenic, downstream) using the software ChIPseeker with GRCh38/hg38 as the reference genomes (58).

ChIP for H3K4me3, H3K27ac, and H3K27me3 in H1155, H526, A549, and H460 was performed as above. Results were quantified by real-time PCR with SYBR green dye using the ABI Prism 7900 system (Applied Biosystems). Primers used in this study are listed in Supplemental Table 11.

### RNA-Seq.

Total RNA was isolated by TRIzol reagent (Invitrogen) and mRNA was purified using poly-T oligo-attached magnetic beads. The RNA was randomly fragmented by using divalent cations. Then, the RNA fragments were reverse-transcribed into cDNA, which were end-repaired, A-tailed at the 3′ end, and adaptor ligated. The quality and quantity of each cDNA library were tested by Bioanalyzer 2100 system (Agilent). According to commercial protocols, the cDNA libraries were sequenced by the NovaSeq 6000 system (Illumina). Quality control was performed using FastQC v0.11.8. Clean reads were aligned to the GRCh38/hg38 reference genome using Hisat2 v2.0.5 (59). FPKM (fragments per kilobase per million mapped fragments) counts were estimated using featureCounts v1.5.0-p3, and differential gene expression analysis was performed using DESeq2 v1.20.0 (60) with adjusted *P* ≤ 0.05 and |log_2_ fold-change| ≥ 0. The up- and downregulated genes were used for GO term enrichment analysis. GO term enrichment analyses were performed using Metascape database (http://metascape.org/), and GSEA was conducted using clusterProfiler package v3.18.1 (61).

### GST pulldown assay.

The Ras-binding domain (RBD) of RalGDS (aa 788-914) and Raf1 (aa 51-131) can serve as an activation-specific probe for Rap1 and Ras. RBDs of RalGDS and Raf1 were cloned into pGEX-4T vector for expression with an N-terminal GST tag. GST-Rap1-RBD and GST-Raf1-RBD proteins were expressed at 16°C in BL21(DE3) cells. For GST pulldowns, BL21(DE3) cells were lysed by sonication in PBS containing 1% Triton X-100 and 1 mM PMSF. After centrifugation, the supernatant was purified on glutathione (GSH) Sepharose beads (GE Healthcare) in PBS containing 1% Triton X-100 and 1 mM PMSF.

A549 and H1155 cells were lysed by RIPA lysis buffer (150 mM NaCl, 50 mM Tris-HCl pH 7.4, 1% NP-40, 1% deoxycholic acid, 1 mM EDTA, 5% glycerol, 1 M DTT, 2 mM PMSF). Lysis was performed at 4°C for 60–90 minutes. Lysates were clarified by centrifugation at maximal speed in an Eppendorf centrifuge for 10 minutes at 4°C. GSH Sepharose beads were added to the supernatant and incubated at 4°C for 6–8 hours with slight agitation. Beads were washed 4 times in RIPA. After the final wash, Laemmli sample buffer was added to the samples. Next, proteins were fractionated by SDS-PAGE and transferred to PVDF membranes (MilliporeSigma). Monoclonal antibodies against Rap1 (Abcam) and Ras (Abcam) were used specifically to detect Rap1 and Ras. Immune complexes were detected by chemiluminescence (Amersham).

### Chromosome conformation capture.

Chromosome conformation capture (3C) was performed as described previously (62). *Dpn* II was used as a restriction endonuclease site for fragmentation. The PCR product from the *RASGRP2* gene promoter to enhancer was amplified, digested with *Dpn* II, and ligated at high concentrations to generate all possible ligation products. The cross-linking and ligation efficiencies between different samples were normalized by setting the highest cross-linking frequency to 1.0. Primers used in this study are provided in Supplemental Table 11.

### Luciferase assay.

The DNA fragment of the *RASGRP2* promoter amplified from HUVEC DNA was inserted into the *Bgl* II site of the polylinker region pGL3-basic by using homologous recombination. The *Bgl* II site in the reverse recombination primer of the *RASGRP2* promoter was not complete to prevent cleavage during the fragmentation of pGL3-basic containing the DNA fragment of the *RASGRP2* promoter. The DNA single strand of the *RASGRP2* enhancer was synthesized, annealed, and inserted into the *Bgl* II site of pGL3-basic containing the DNA fragment of the *RASGRP2* promoter. HEK293 cells were transiently cotransfected with pRL-CMV Renilla luciferase reporter, which was used for normalization. Cells were harvested and assayed for luciferase activity using dual luciferase reporter assay systems (Promega) following the manufacturer’s instructions at the time of 24 hours after transfection.

### Public domain single-cell RNA-Seq data sets.

We used single-cell RNA-Seq data sets of human lung cancers generated by Rudin and colleagues (19). The single-cell RNA-Seq dataset from Rudin and colleagues was previously imputed from their study using the SEQC pipeline. We utilized Seurat suite version 3.1 (63) to perform cell clustering. Cells were clustered and visualized after the dimension reduction of UMAP (uniform manifold approximation and projection).

Differentially expressed genes between 2 groups of cells were identified using a Wilcoxon rank-sum test by function FindMarkers of Seurat. As for individual clusters, differentially expressed genes were detected against the average expression of all other clusters. The log-fold changes of all genes were also calculated by FindMarkers and GSEA analysis. GSEA analysis was performed by function gseGO of the ClusterProfiler package (v3.18.1) (61) using GO Biological Process ontology gene sets.

### Data availability.

The RNA-Seq data of control and GFI1-expressing A549 cells are in NCBI’s Gene Expression Omnibus (GEO) under accession number GSE165307. The accession number for the RNA-Seq data of WT and GFI1-KO H1155 cells is GSE165308. The ChIP-Seq data are deposited under GEO accession number GSE164984.

### Statistics.

An unpaired 2-tailed Student’s *t* test was used to compare the means of 2 populations. Multiple comparisons were performed with a 1-way ANOVA test with post hoc contrasts by Tukey’s test. A *P* value less than 0.05 was considered statistically significant for all tests.

### Study approval.

All animal procedures were approved by the IACUC at Tianjin Medical University (TMUaMEC2021058) and conformed to the legal mandates and national guidelines for the care and maintenance of laboratory animals. Human lung cancer tissues were collected at Tianjin Medical University Cancer Hospital. The use of all human lung cancer specimens was approved by the IRB of Tianjin Medical University Cancer Hospital (bc2020178). Written informed consent was obtained from all patients, and samples were deidentified prior to analysis.

## Author contributions

Z Liu, HW, and ZM designed the study. HW, Z Lin, ZN, WZ, WL, and FY performed experiments and analyzed the results. XW, ZX, and ZZ provided lung cancer specimens. Z Liu wrote the manuscript. HW and Z Lin are co–first authors. HW is listed first because he led the experiments.

## Supplementary Material

Supplemental data

Supplemental table 1

Supplemental table 2

Supplemental table 3

Supplemental table 4

Supplemental table 5

Supplemental table 6

Supplemental table 7

Supplemental table 8

Supplemental table 9

Supplemental table 10

Supplemental table 11

## Version 1 07/12/2022

In-Press Preview

## Version 2 09/01/2022

Electronic publication
