# Supplementary material for: Hematopoietic transcription factor GFI1 promotes anchorage independence by sustaining ERK activity in cancer cells
Source: J Clin Invest. 2022 Sep 1;132(17):e149551. doi: 10.1172/JCI149551 (PMC9433100; doi:10.1172/JCI149551)

Hematopoietic transcription factor GFI1 promotes anchorage independence by sustaining ERK activity in cancer cells

Hao Wang[1#], Zhenzhen Lin[1#], Zhe Nian[1], Wei Zhang[1], Wenxu Liu[1], Fei Yan[1], Zengtuan Xiao[1,4], Xia Wang[5], Zhenfa Zhang[4], Zhenyi Ma[1]*, and Zhe Liu[1,2,3]*

[1]State key laboratory of Experimental Hematology, Haihe Laboratory of Cell Ecosystem, The Province and Ministry Co-sponsored Collaborative Innovation Center for Medical Epigenetics, Key Laboratory of Immune Microenvironment and Disease of the Ministry of Education, Department of Immunology, School of Basic Medical Sciences, Tianjin Medical University

[2]Department of Cell Biology, School of Basic Medical Sciences, Hangzhou Normal University, Hangzhou, China

[3]Collaborative Innovation Center for Cancer Personalized Medicine, Nanjing Medical University, Nanjing, China

[4]Department of Lung Cancer Center, Tianjin Medical University Cancer Institute and Hospital, Tianjin, China

[5]Department of Gastroenterology, Tianjin Medical University Cancer Institute and Hospital, Tianjin, China

A

	sample	percentage of *GFI1* -expressing cells in tumor cells
NSCLC	Ru653	8.70%
	Ru661	1.80%
	Ru675	0.91%
	Ru676	0.83%
	Ru682	0.86%
	Ru684	0.76%
	Ru1170	0.78%
	Ru1128	1.96%
	Ru669	0.00%
	Ru1057	0.00%
	Ru1134	0.00%
	Ru1135	0.00%
	Ru1137	0.00%
	Ru1141	0.00%
SCLC	RU426	6.22%
	RU1066	0.40%
	RU1145	6.60%
	RU1229	7.01%

B

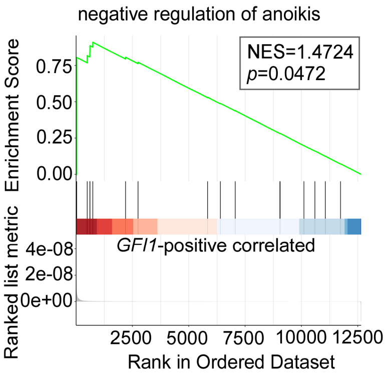

Supplemental Figure 1. **Analysis of single-cell RNA-Seq datasets of human lung cancers generated by Rudin and colleagues.** (**A**) The percentage of *GFI1*-expressing cells in 8 out of 14 human primary NSCLCs and all 4 human primary SCLCs by single-cell RNA-Seq in HTAN Data Portal. (**B**) GSEA shows that the *GFI1*-expressing cancer cells in NSCLC exhibited upregulated negative regulation of anoikis.

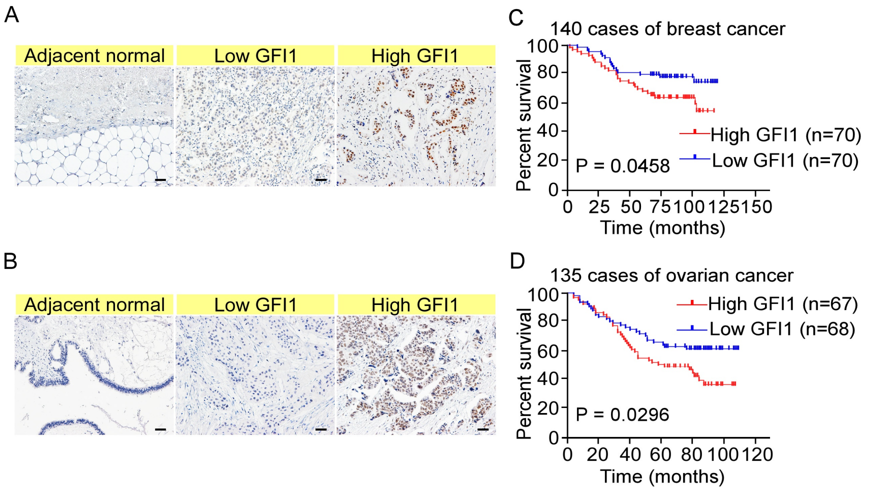

Supplemental Figure 2. **GFI1 is expressed in breast and ovarian cancer cells and predicts poor prognosis.** (**A**) IHC staining with anti-GFI1 was performed on 10 normal breast tumor adjacent tissues and 140 breast cancer specimens. Scale bars represent 50 µm. (**B**) IHC staining with anti-GFI1 was performed on 2 normal ovarian tumor adjacent tissues and 135 ovarian cancer specimens. Scale bars represent 50 µm. (**C**) Kaplan-Meier survival rates for 140 subjects with breast cancer with low versus high GFI1 expression were compared. (**D**) Kaplan-Meier survival rates for 135 subjects with ovarian cancer with low versus high GFI1 expression were compared.

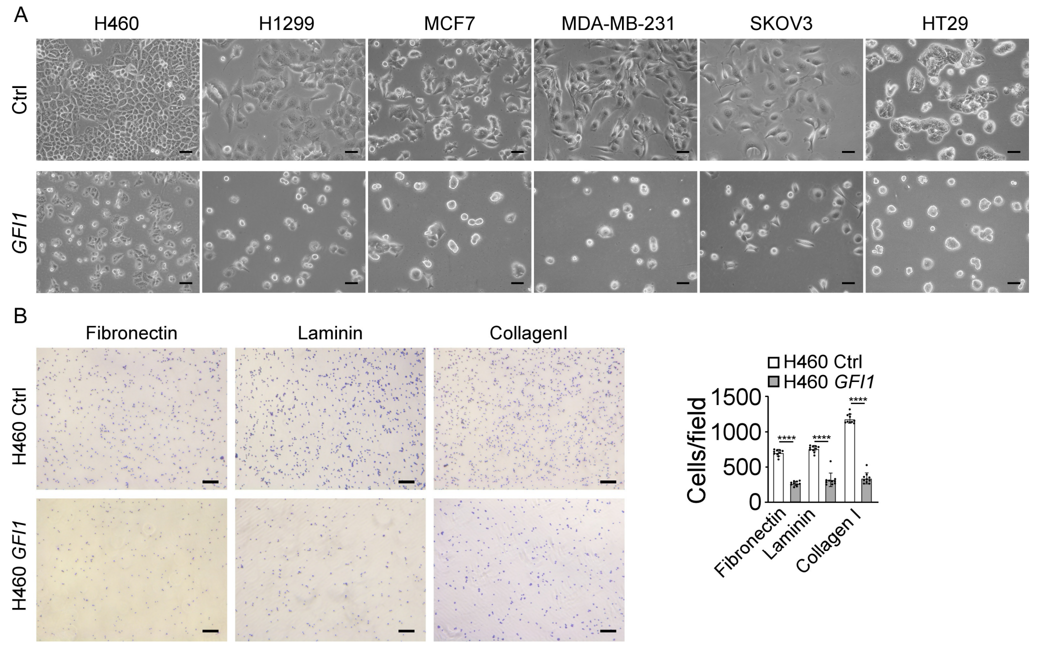

Supplemental Figure 3. **GFI1 can promote cell detachment in various cell lines.** (**A**) Morphology of H460, H1299, MCF-7, MDA-MB-231, SK-OV-3, HT-29 cells expressing either control vector or GFI1. Scale bars represent 40 µm. (**B**) GFI1-expressing H460 cells were plated on fibronectin-coated, laminin332-coated and collagen I-coated plates. After 15 min, attached cells were counted. Scale bars represent 200 µm. Bar graph below shows the number of adherent cells. Mean ± SD represents 10 visualized areas in one experiment. Three independent experiments were performed. ****, $P < 0.0001$ (unpaired two-tailed Student's t-test).

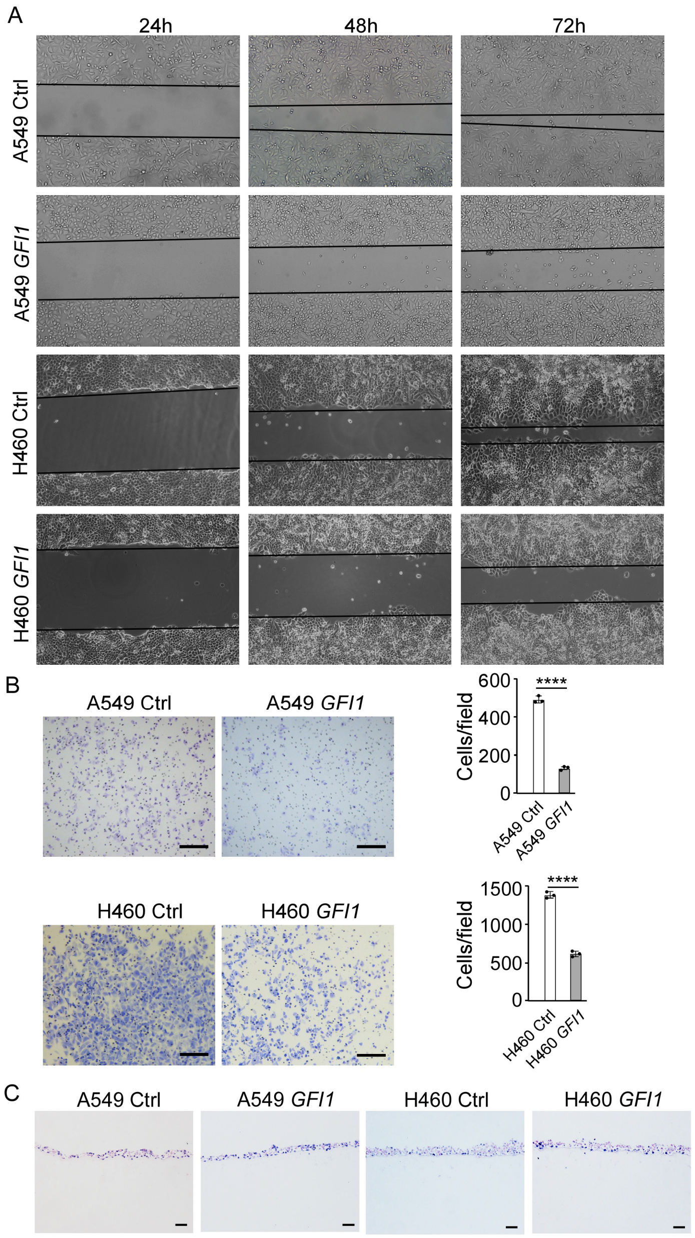

Supplemental Figure 4. GFI1 can suppress migration and matrigel invasive capability of cancer cells. (**A**) Images of wound-healing assay showed the motility of A549, *GFI1*-expressing A549, H460 and *GFI1*-expressing H460 cells. (**B**) Control and *GFI1*-expressing cancer cells were subjected to a transwell assay. Error bars, means ± SD for a representative experiment performed in triplicate. Scale bars represent 100 µm. ****, $P < 0.0001$ (unpaired two-tailed t test). (**C**) Representative H&E-stained sections of A549 and H460 cells after *GFI1* overexpression on 3D dense gel. The experiment performed in triplicate. Scale bars represent 100 µm.

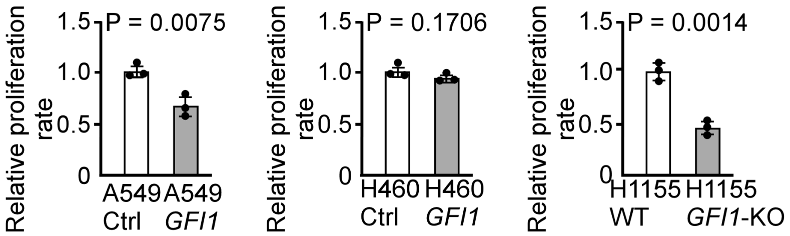

Supplemental Figure 5. **BrdU incorporation assay for indicated cells.** Mean ± SD represents 3 replicates in one experiment. Three independent experiments were performed (unpaired two-tailed Student's t-test).

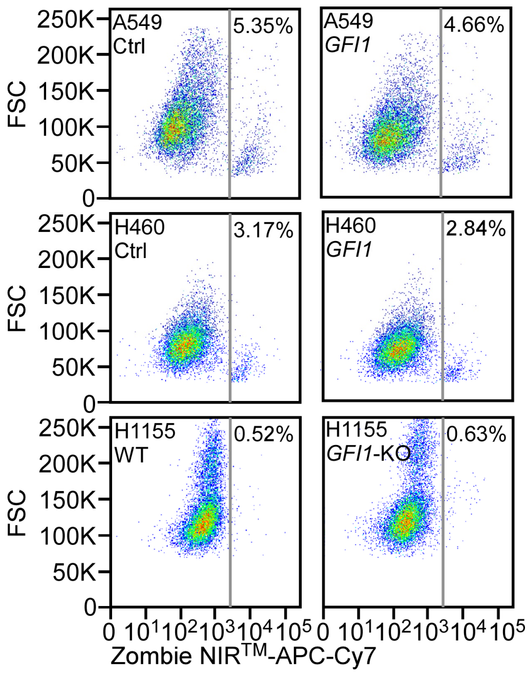

Supplemental Figure 6. **All studied cells exhibit good viability.** Before adhesion, migration and invasion assay, cells were stained with Zombie NIR™, an amine reactive fluorescent dye that is non-permeant to live cells. FACS analysis shows that all studied cells exhibit good viability.

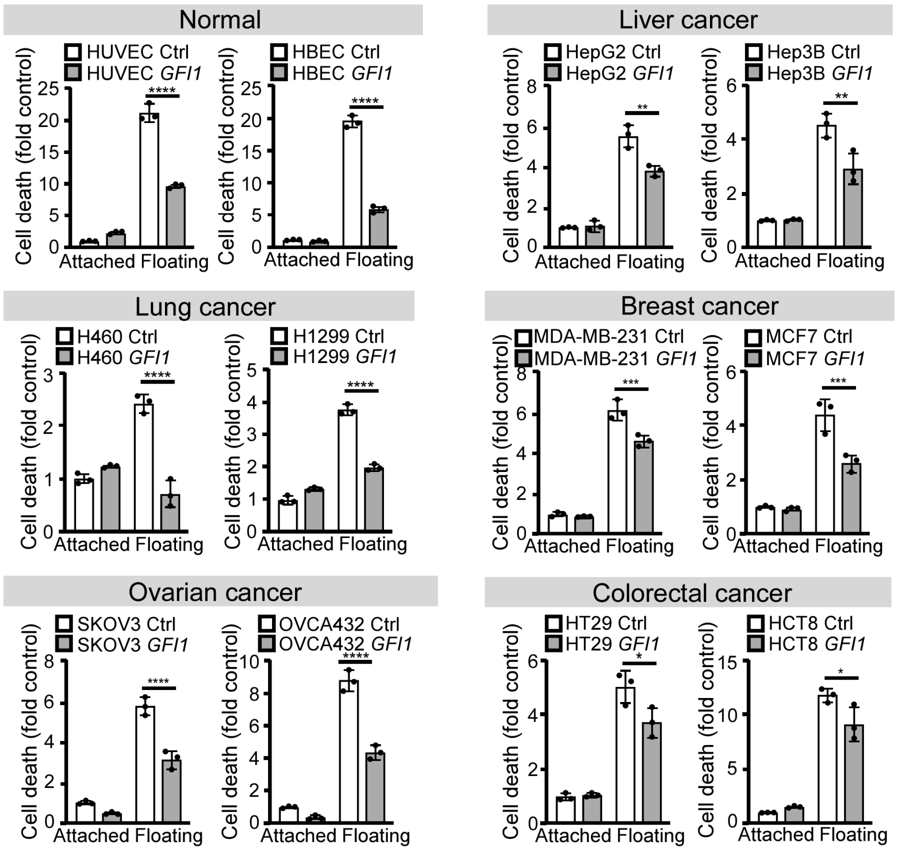

Supplemental Figure 7. **GFI1 can promote anchorage independence in various cell lines.** Cell death of *GFI1*-expressing normal cells and cancer cells were assessed after 16 hr and 24 hr under attached or floating conditions. Mean ± SD represents 3 replicates in one experiment. Three independent experiments were performed. Error bars represent SEM. *, $P < 0.05$; **, $P < 0.01$; ***, $P < 0.001$; ****, $P < 0.0001$(one-way ANOVA test with post hoc contrasts by Tukey's test).

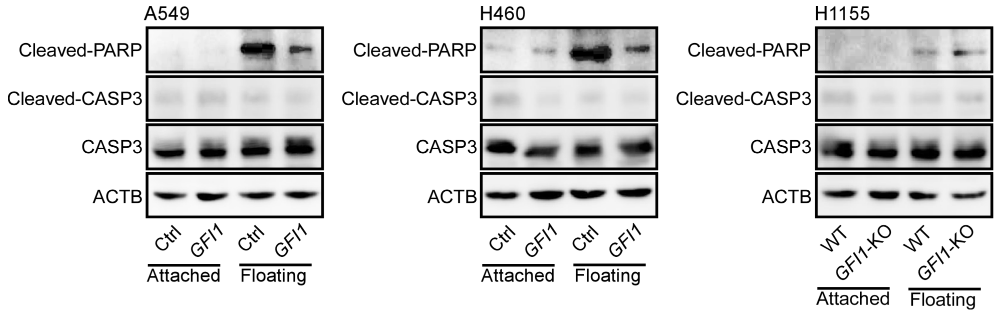

Supplemental Figure 8. Immunoblot showing expression of Cleaved-PARP, Cleaved-CASP3, CASP3 and ACTB. See complete unedited blots in the supplemental material.

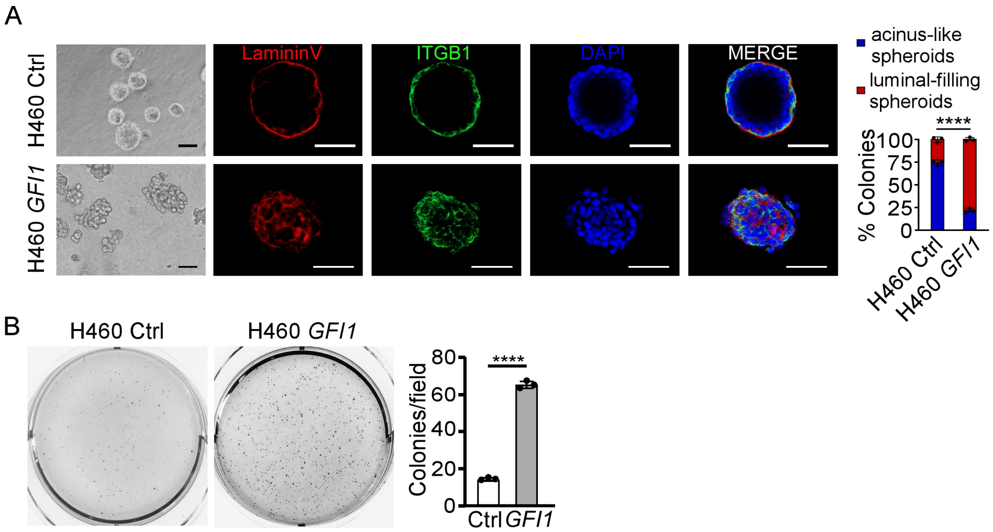

Supplemental Figure 9. GFI1 promotes cell detachment and anchorage independence in H460 cells. (**A**) H460 and *GFI1*-expressing H460 cells were cultured in Matrigel for 8 days. Confocal midpoint slices of acinus stained for ITGB1, LamininV, and DAPI are shown. Colonies > 50 µm in diagram were counted. Scale bars represent 50 µm. Three independent experiments were performed. Error bars represent SEM. ****, P < 0.0001 (unpaired two-tailed Student's t-test). (**B**) Indicated cells were allowed to grow in soft agar for 2 weeks, and colonies were counted. Error bars, means ± SD for a representative experiment performed in triplicate. Three independent experiments were performed. ****, P < 0.0001 (unpaired two-tailed Student's t-test).

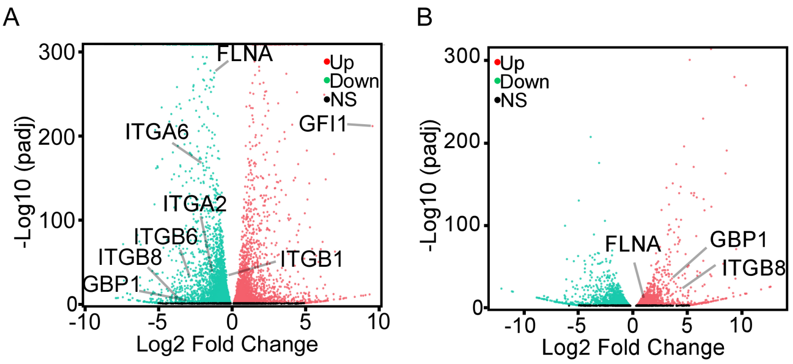

Supplemental Figure 10. The volcano plot maps based on RNA-Seq data. (**A**) Changes of the gene expression profile in A549 cells with *GFI1* expression. (**B**) Changes of the gene expression profile in H1155 cells upon *GFI1* deletion.

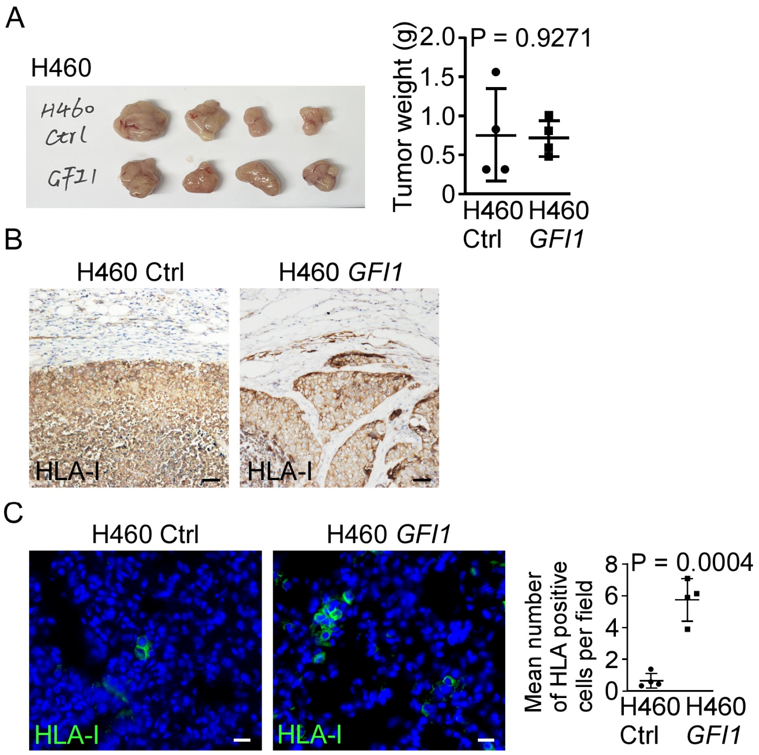

Supplemental Figure 11. GFI1 enhances H460 cell invasiveness and the ability to infiltrate the lung tissue. (**A**) H460 and *GFI1*-expressing H460 cells were mixed with cancer-associated fibroblasts and subcutaneously injected into 8-week-old female BALB/c nude mice. Three weeks later, the mice were sacrificed and analyzed. Subcutaneous tumors are shown. (**B**) IHC staining for HLA-I was performed in the subcutaneous tumor tissue sections. *GFI1* expression shifted the subcutaneous tumors from the expansive to the invasive phenotype. Scale bars represent 50 µm. (**C**) Immunofluoresence for HLA-I was performed in lung tissue sections. The number of HLA-I-positive cells per field was counted in 10 fields from each section, and 10 sections per mouse were used. Mean values of HLA-I-positive cells/field of individual mouse were used for statistical analysis by unpaired two-tailed Student's t-test. Scale bars represent 20 µm.

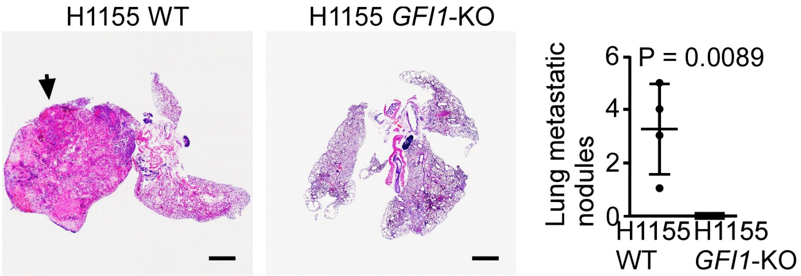

Supplemental Figure 12. GFI1 promotes lung metastasis in a mouse tail vein injection model. Left panel showing H&E staining of lung tissue sections, Scale bars, 2 mm. Right panel showing the quantitation of lung metastatic nodules (unpaired two-tailed Student’s t-test).

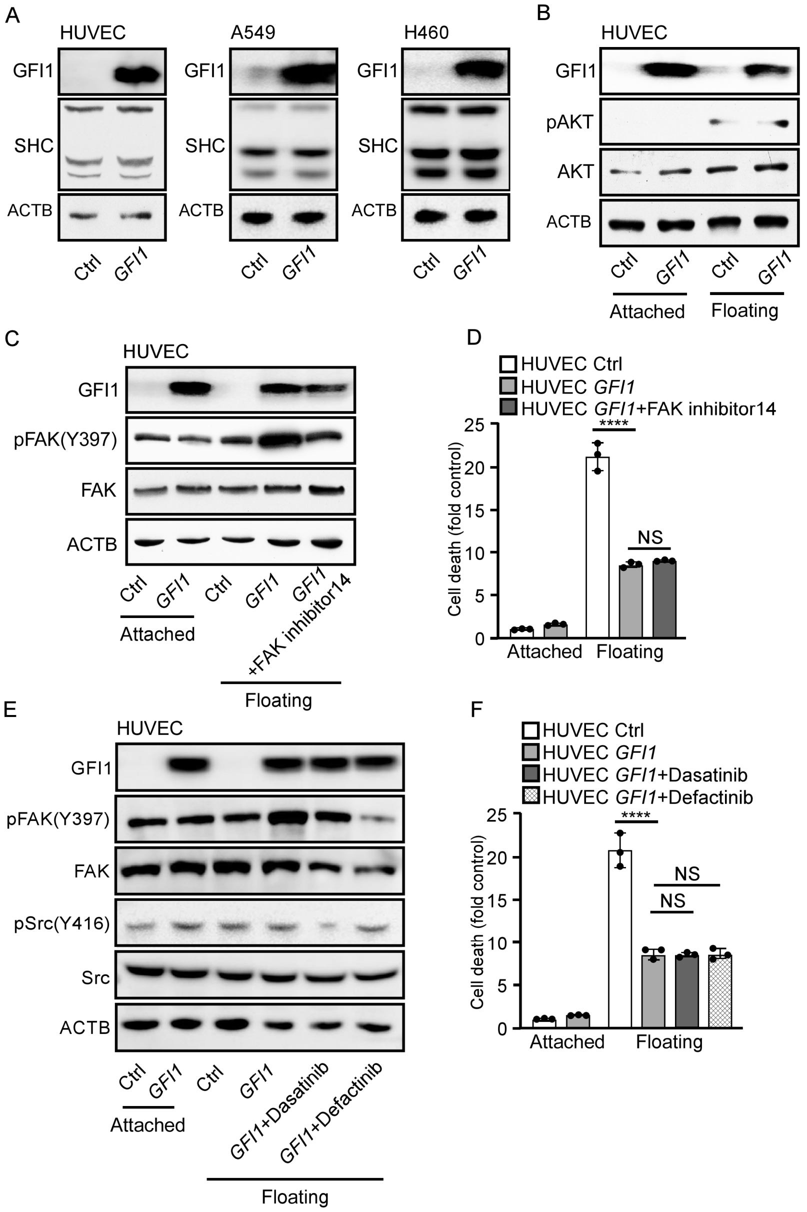

Supplemental Figure 13. p66^{Shc}, AKT and FAK are not involved in GFI1-induced anoikis resistance. (**A**) Immunoblot analysis of SHC level in indicated cells. The blots were generated from the same sample preparation and run at the same time. Three independent experiments were performed. See complete unedited blots in the supplemental material. (**B**) Immunoblot analysis of pAKT level in indicated cells. See complete unedited blots in the supplemental material. (**C**) Immunoblot analysis showed that GFI1 could elevate FAK phosphorylation. See complete unedited blots in the supplemental material. (**D**) The cell death of indicated cells was assessed after cells were under attached or floating condition for 24 hr. Mean ± SD represents 3 replicates in one experiment. Three independent experiments were performed. ****, $P < 0.0001$ (one-way ANOVA test with post hoc contrasts by Tukey's test). (**E**) Immunoblot analysis of pAKT (Y397) and pSrc (Y416) level in indicated cells. See complete unedited blots in the supplemental material. (**F**) The cell death of indicated cells was assessed after cells were under attached or floating condition for 24 hr. Mean ± SD represents 3 replicates in one experiment. Three

independent experiments were performed. ****, $P < 0.0001$ (one-way ANOVA test with post hoc contrasts by Tukey's test).

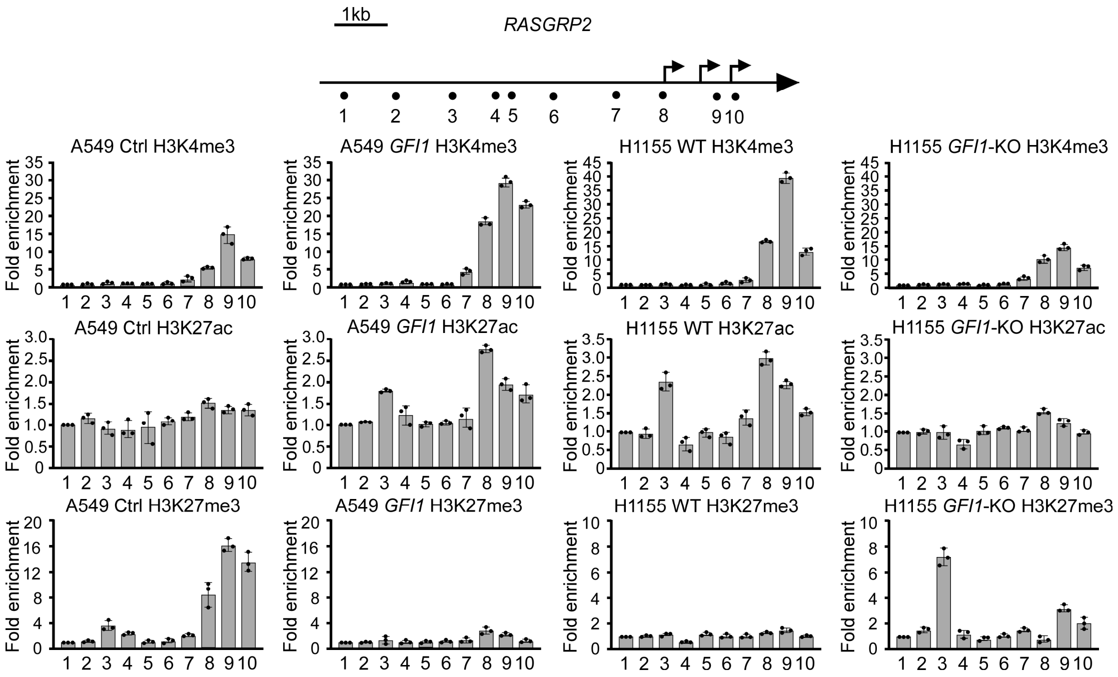

Supplemental Figure 14. ChIP shows distribution of H3K4me3, H3K27ac and H3K27me3 histone modifications in A549, *GFI1*-expressing A549, H1155 and *GFI1*-KO H1155 cells. Location of regions assessed by ChIP is shown in schematic. Error bars represent SD of three independent chromatin preparations.

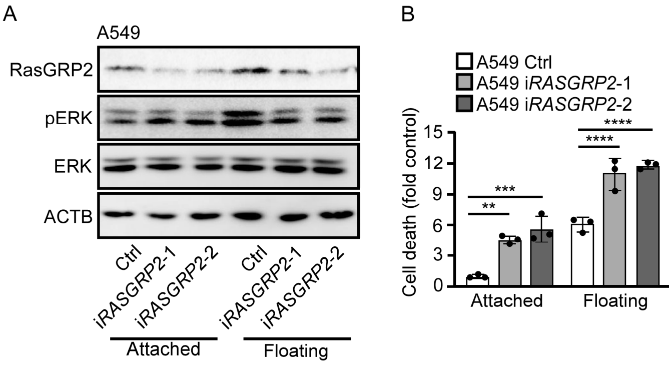

Supplemental Figure 15. Knockdown of RasGRP2 reduces ERK activity in suspended A549 cells and increased cell death in both attached and suspended A549 cells. (**A**) A549 cells were transduced with shRNA against *RASGRP2* and subjected to immunoblot analysis of pERK. See complete unedited blots in the supplemental material. (**B**) The cell death of indicated cells was assessed after 24 hr under attached or floating condition. Mean ± SD represents 3 replicates in one experiment. Three independent experiments were performed. **, $P < 0.01$; ***, $P < 0.001$; ****, $P < 0.0001$ (one-way ANOVA test with post hoc contrasts by Tukey's test).

Full unedited gels for Figure 1C

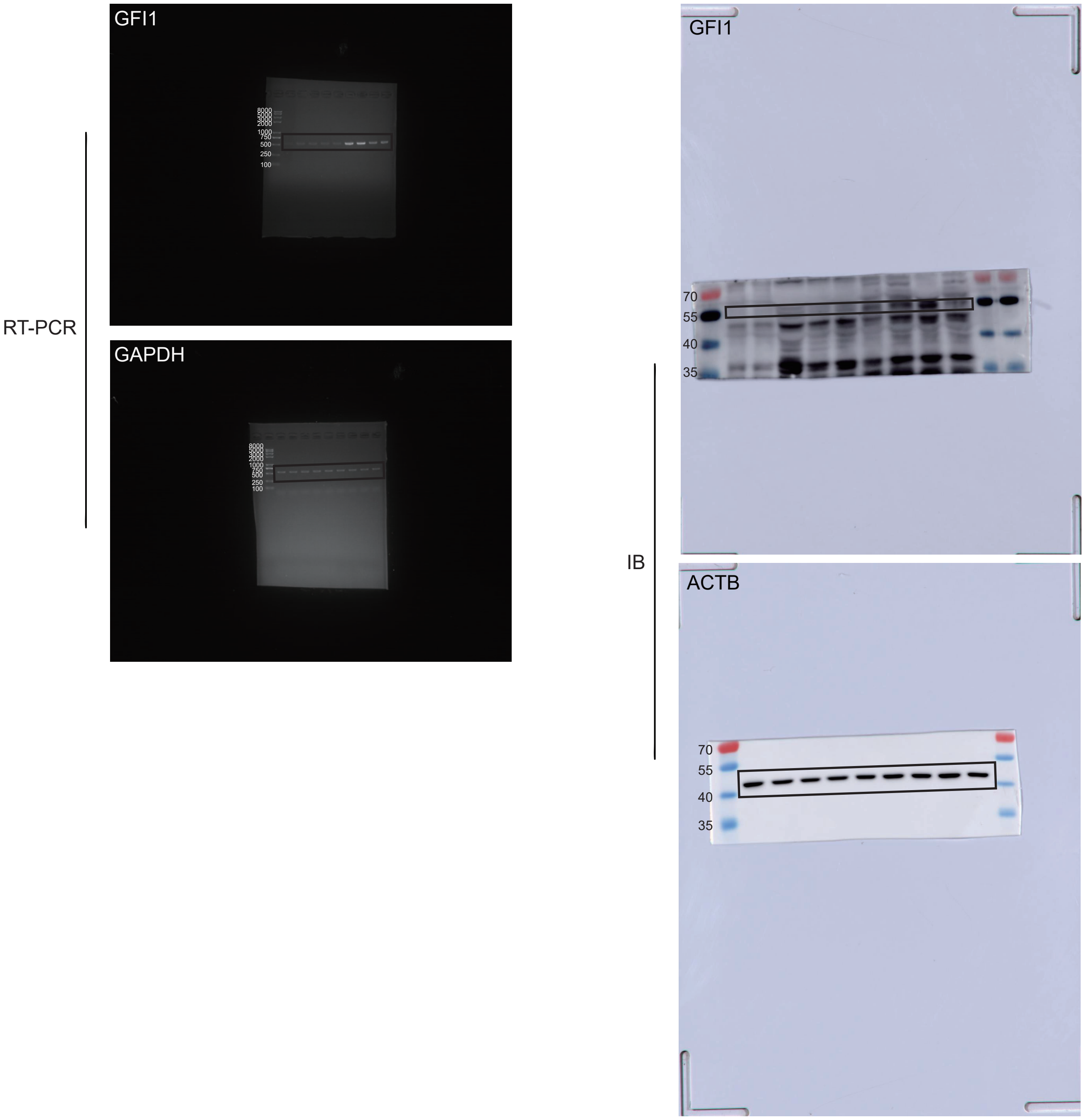

Full unedited gels for Figure 2A

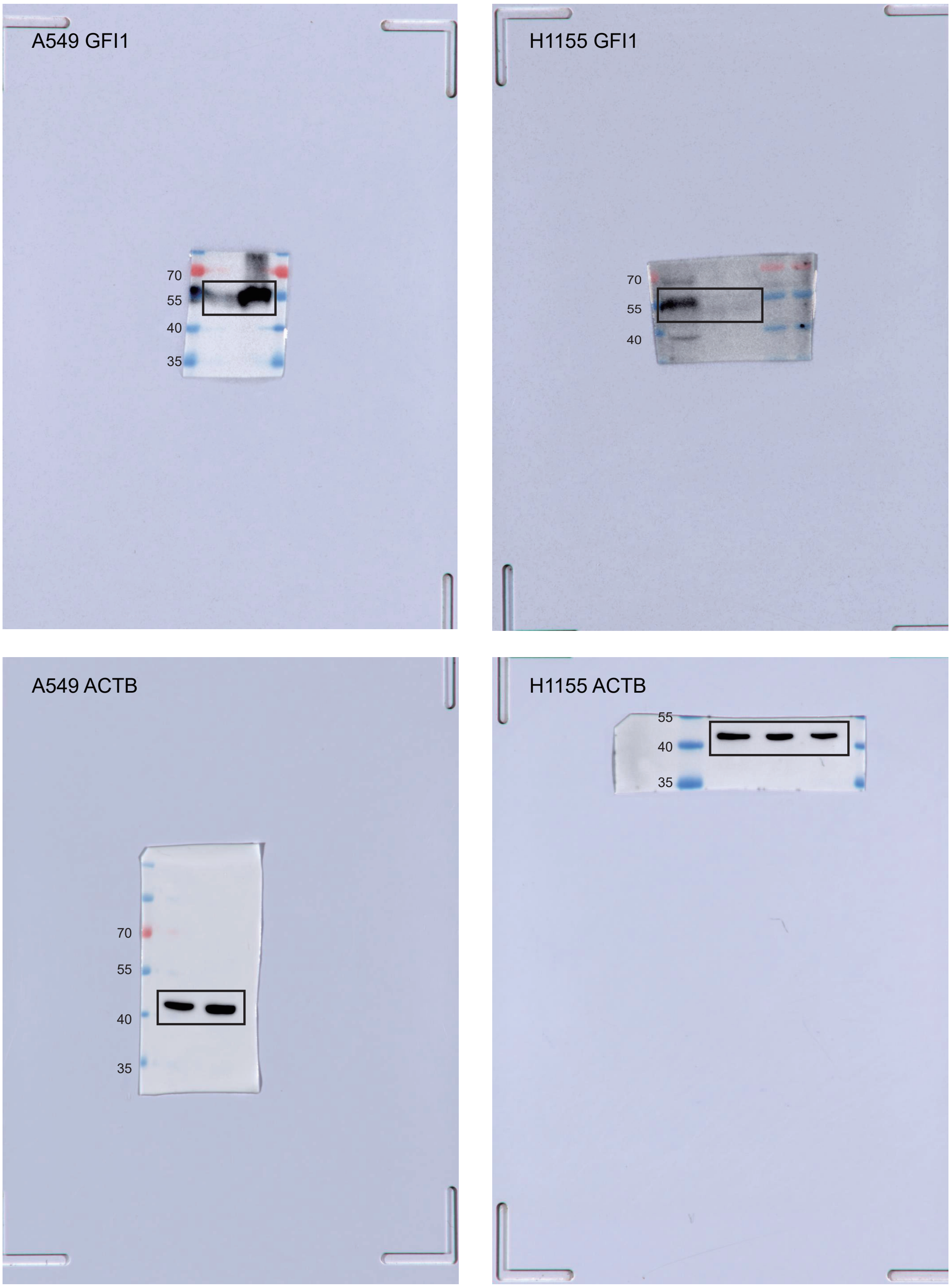

Full unedited gels for Figure 3C

A549

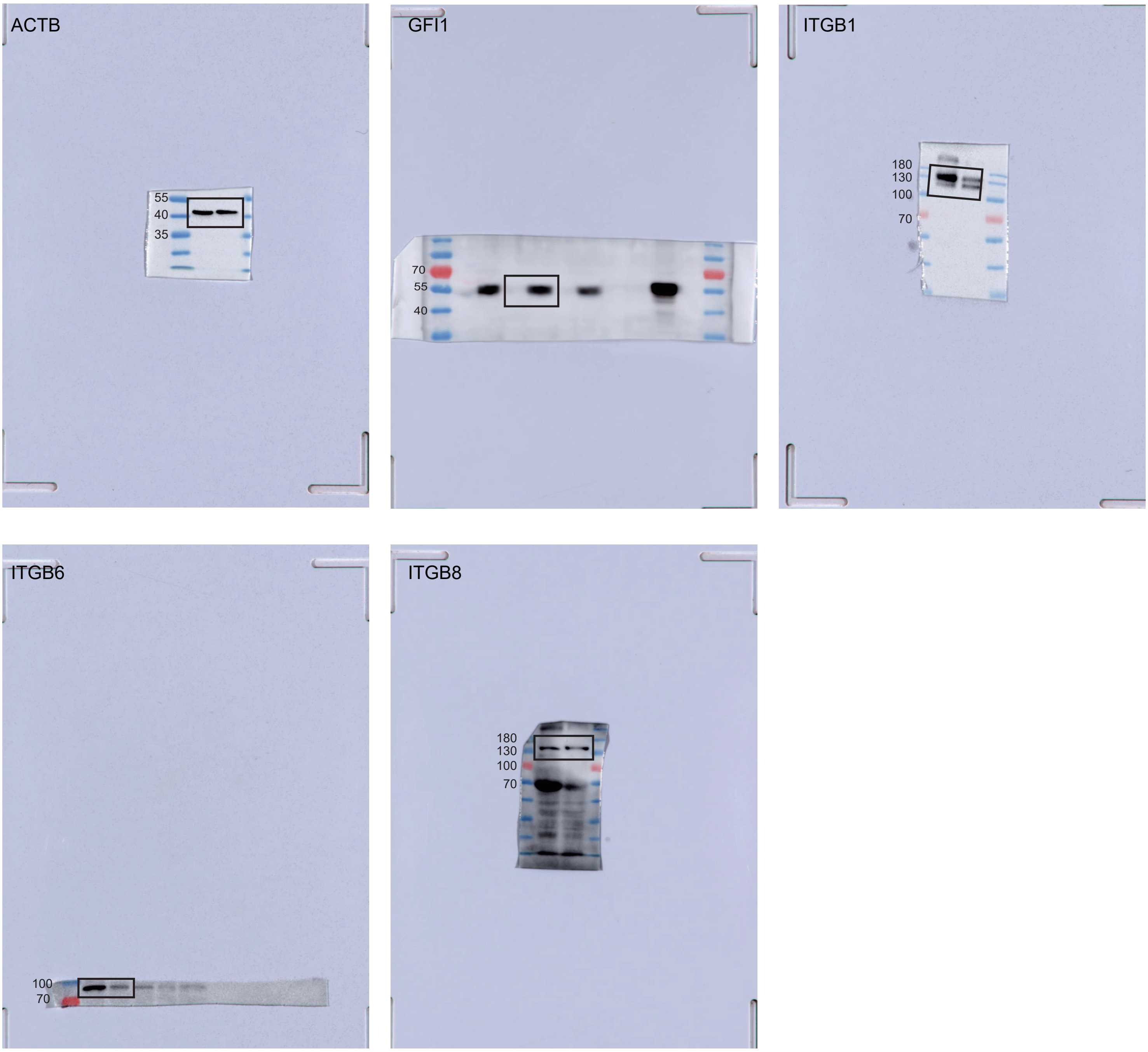

H1155

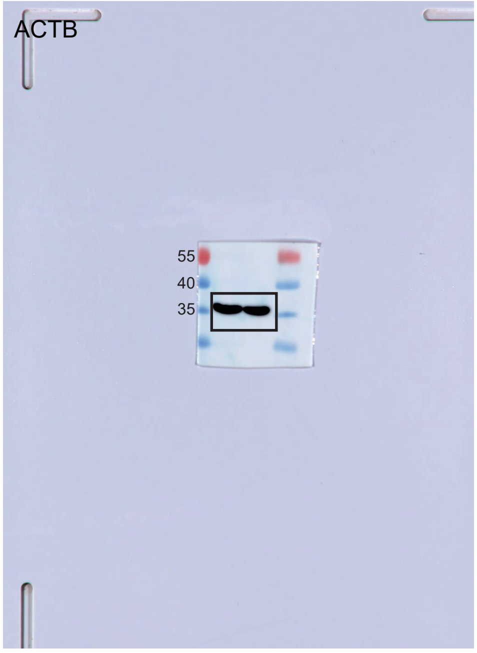
ACTB
55
40
35

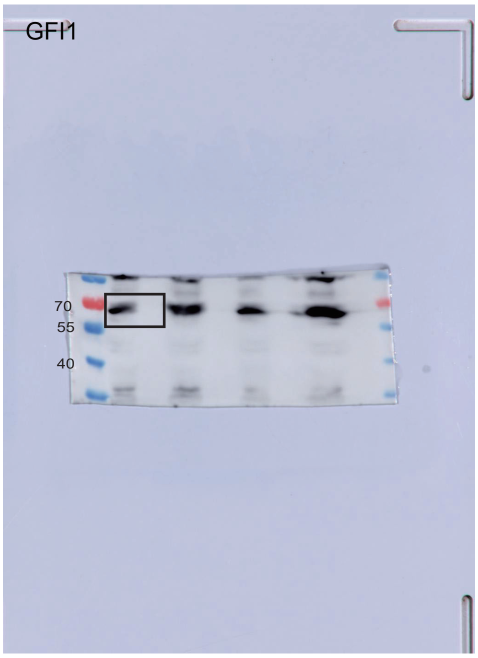
GFI1
70
55
40

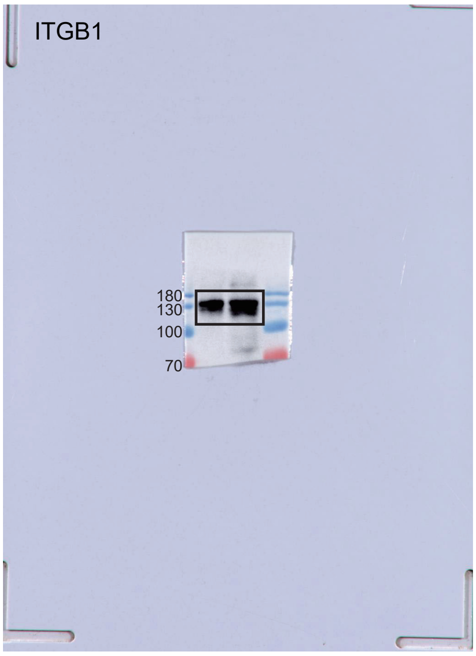
ITGB1
180
130
100
70

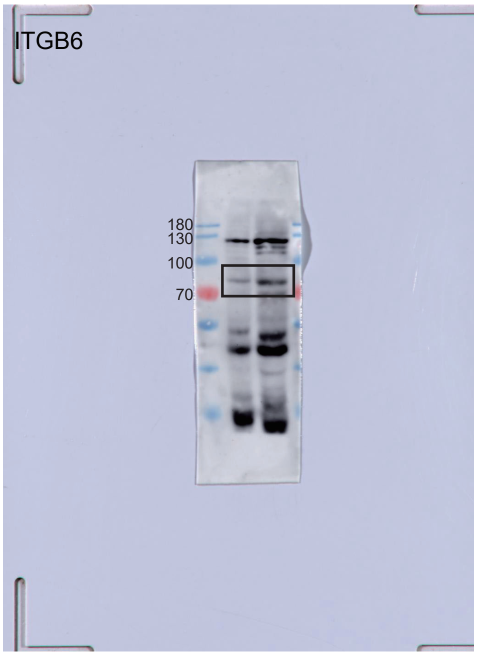
ITGB6
180
130
100
70

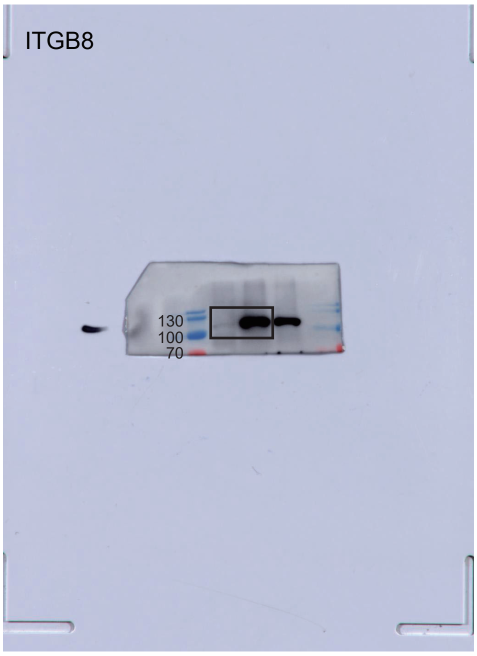
ITGB8
130
100
70

Full unedited gels for Figure 5A

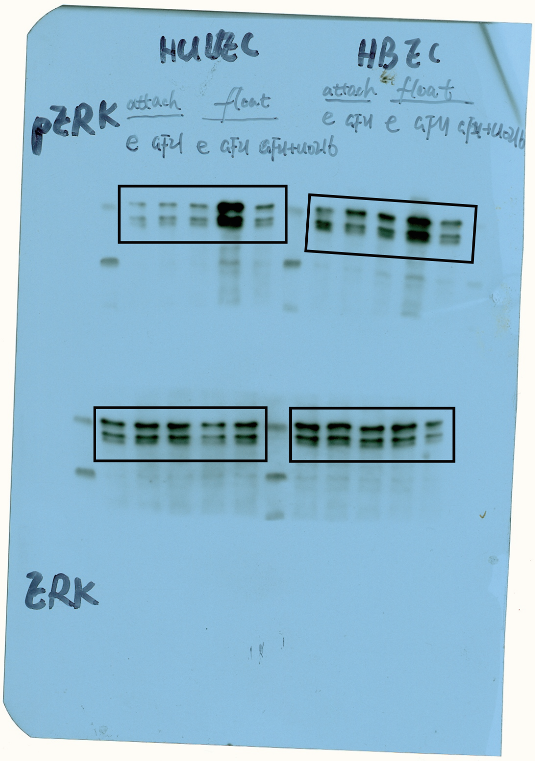

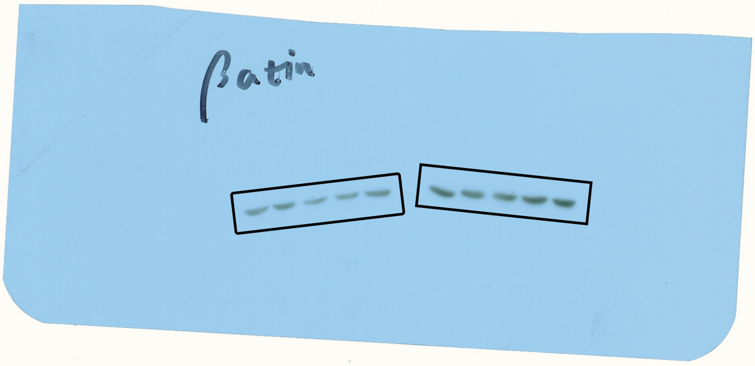

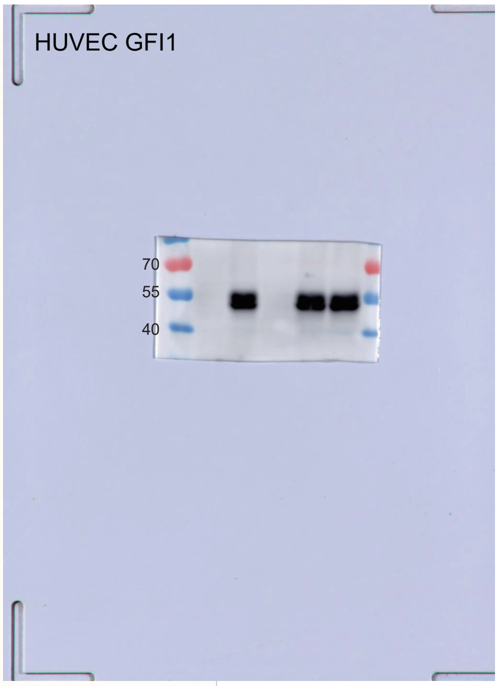

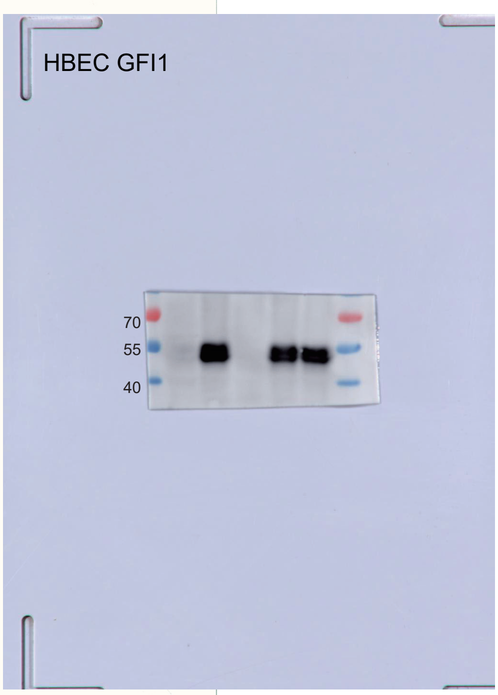

Full unedited gels for Figure 5C

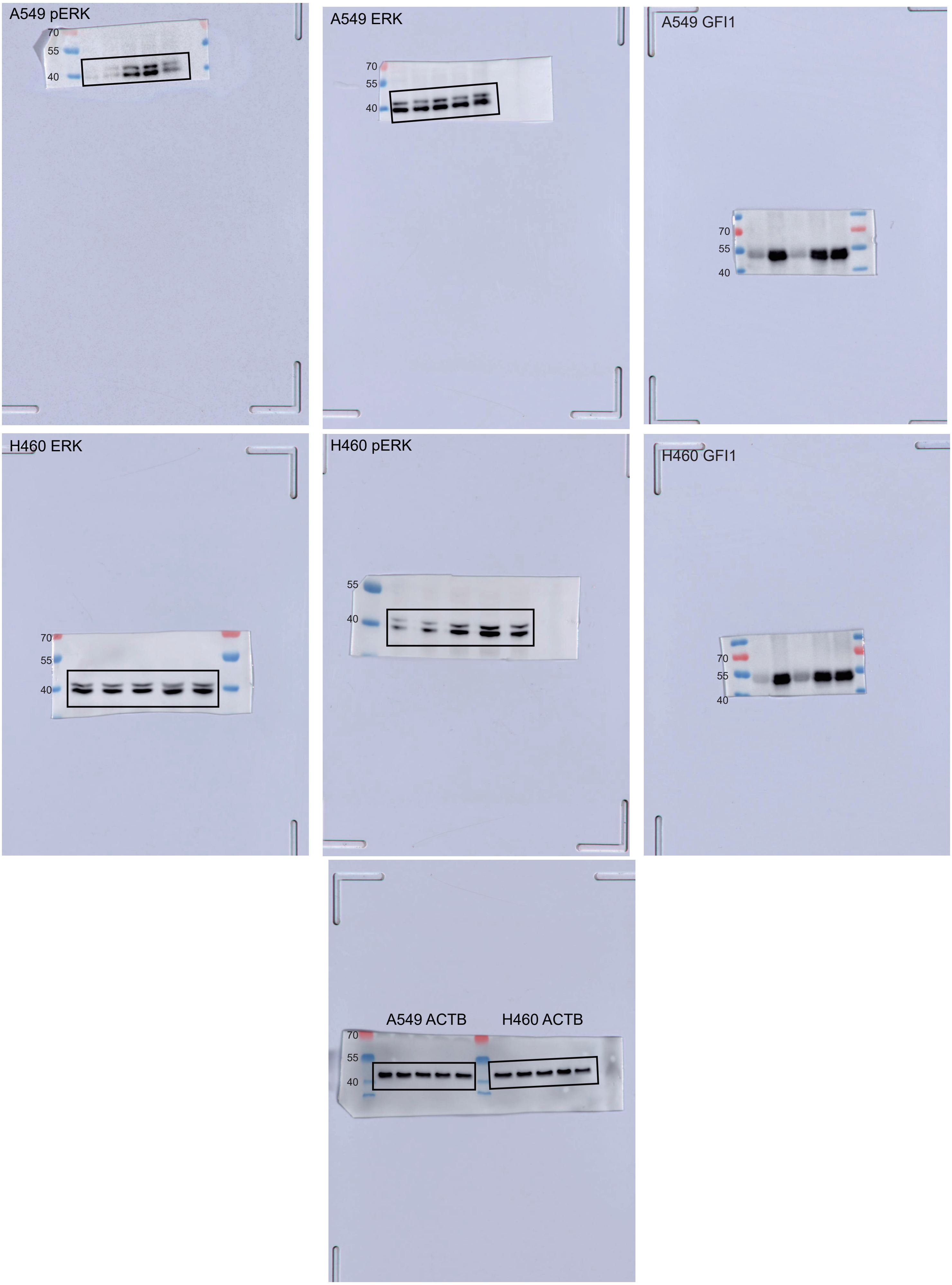

Full unedited gels for Figure 6C

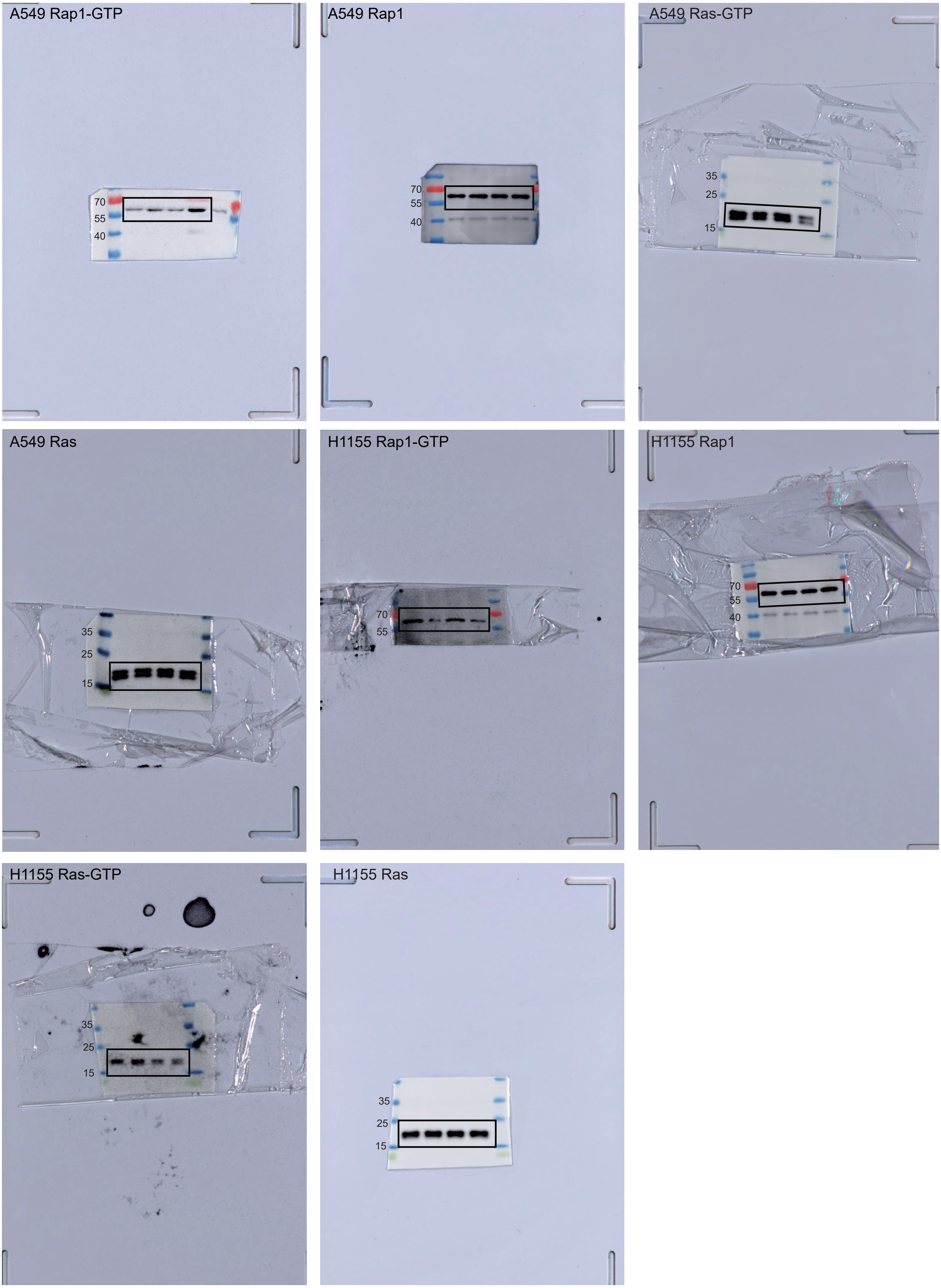

Full unedited gels for Figure 6D

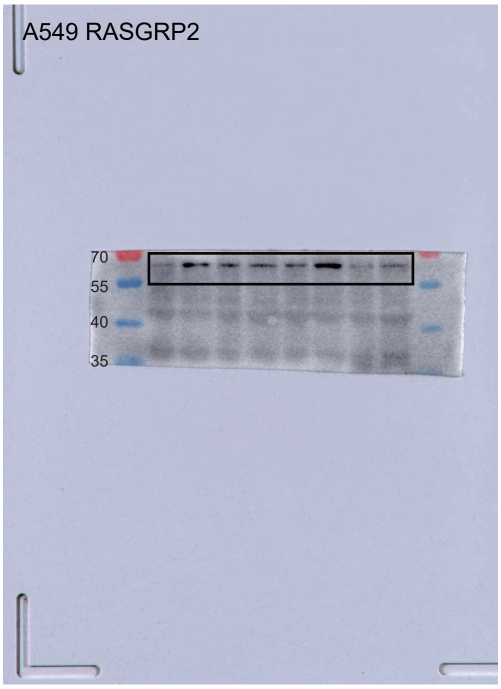
A549 RASGRP2

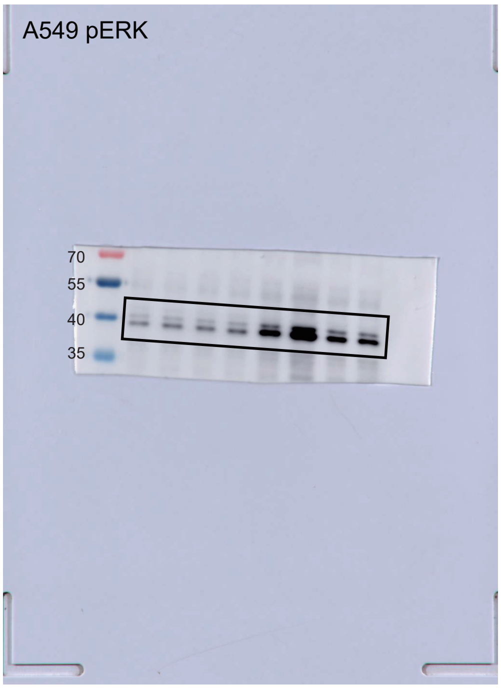
A549 pERK

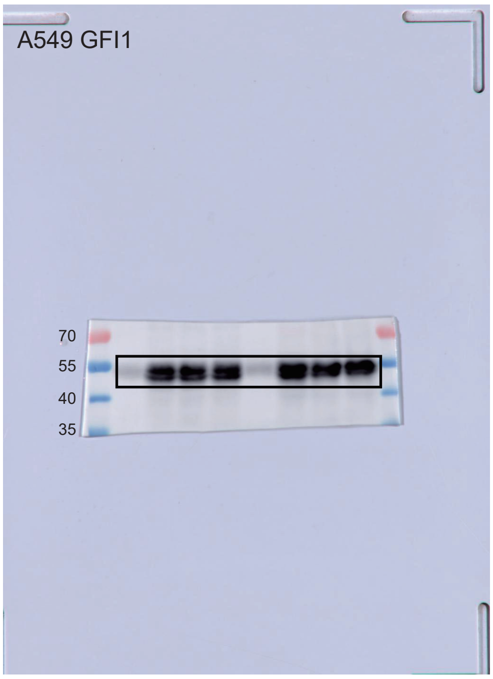
A549 GFI1

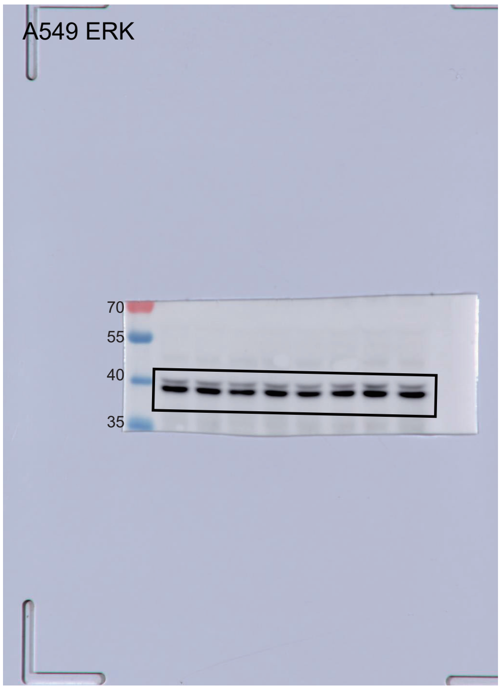
A549 ERK

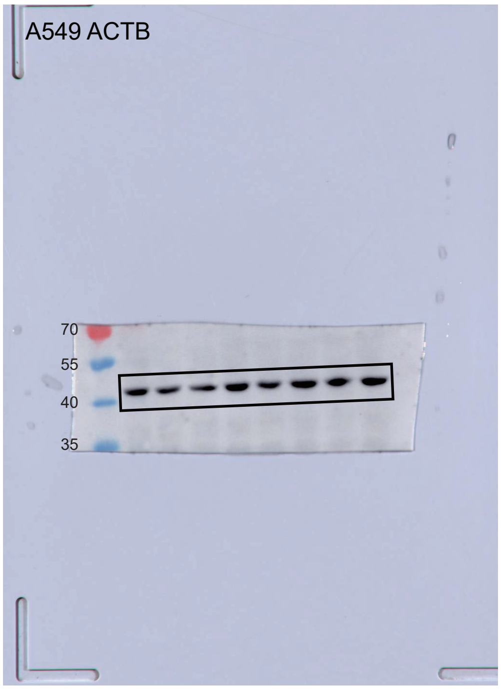
A549 ACTB

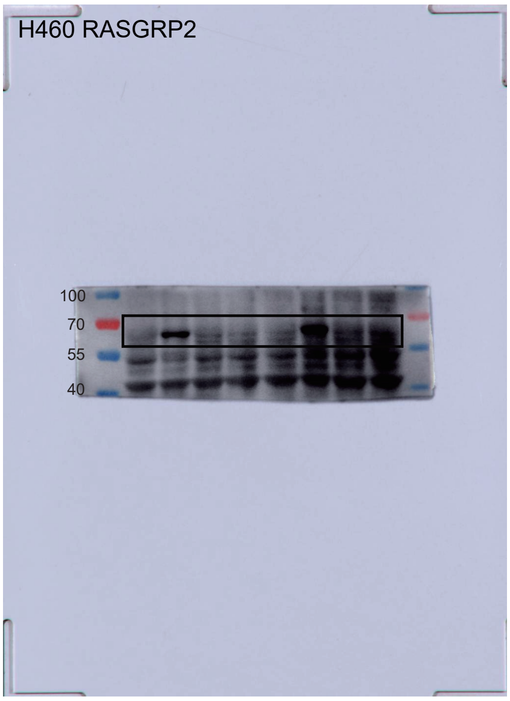
H460 RASGRP2
100
70
55
40

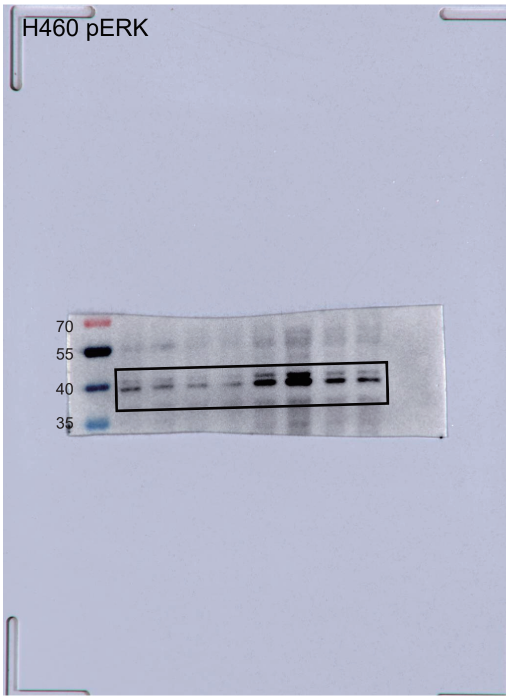
H460 pERK
70
55
40
35

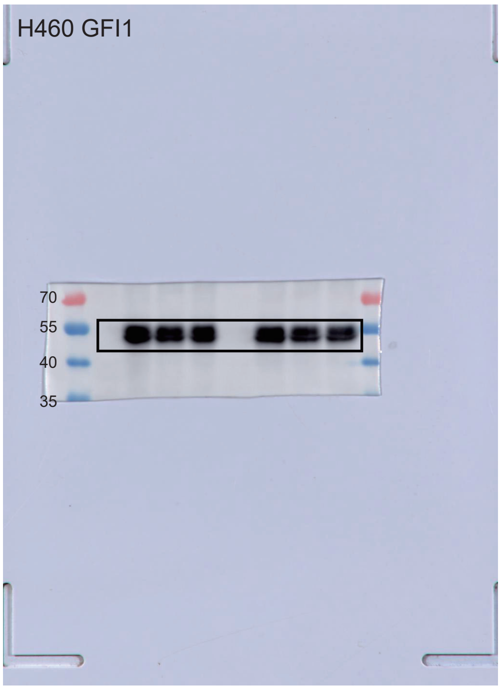
H460 GFI1
70
55
40
35

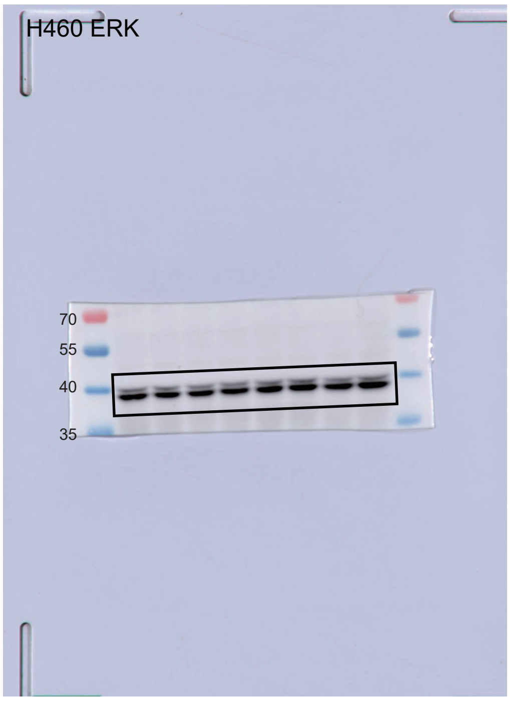
H460 ERK
70
55
40
35

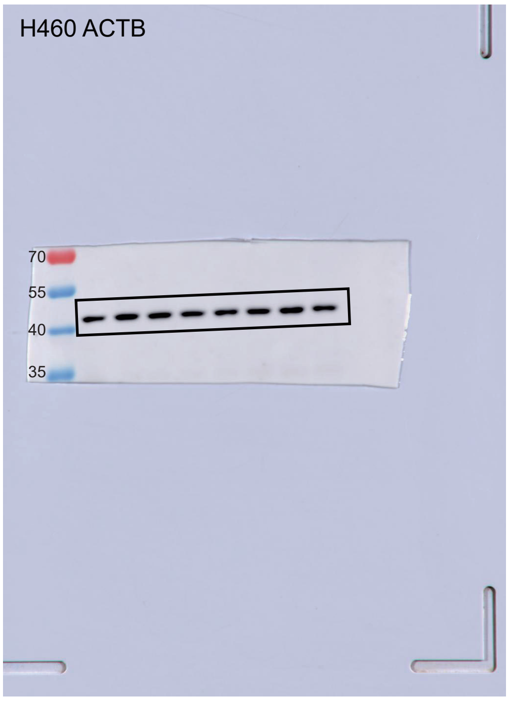
H460 ACTB
70
55
40
35

Full unedited gels for Figure 6F

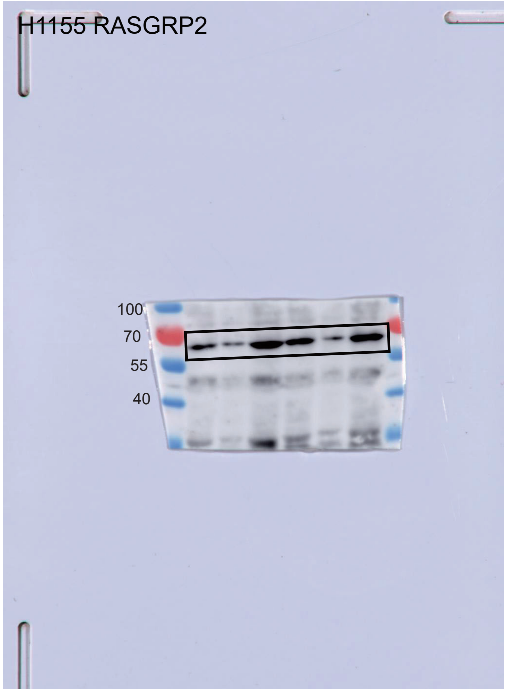

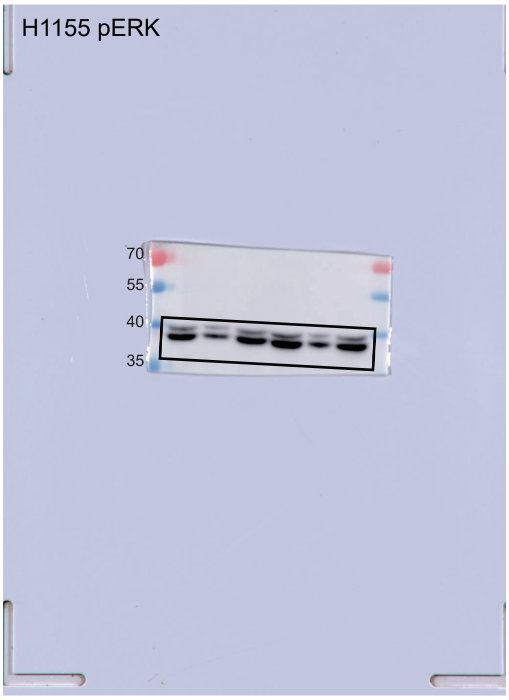

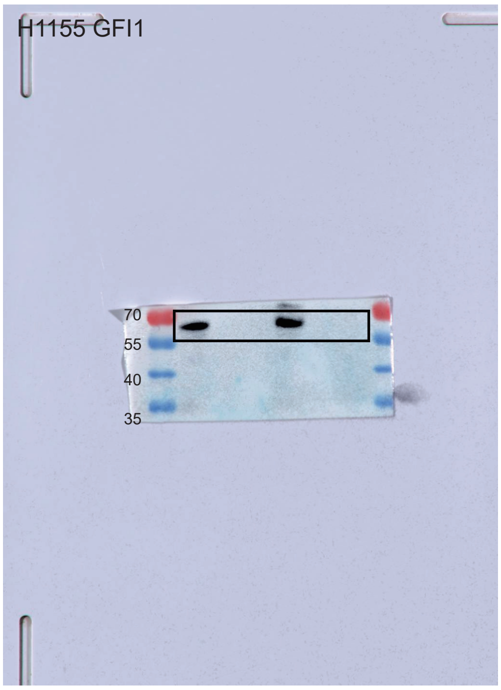

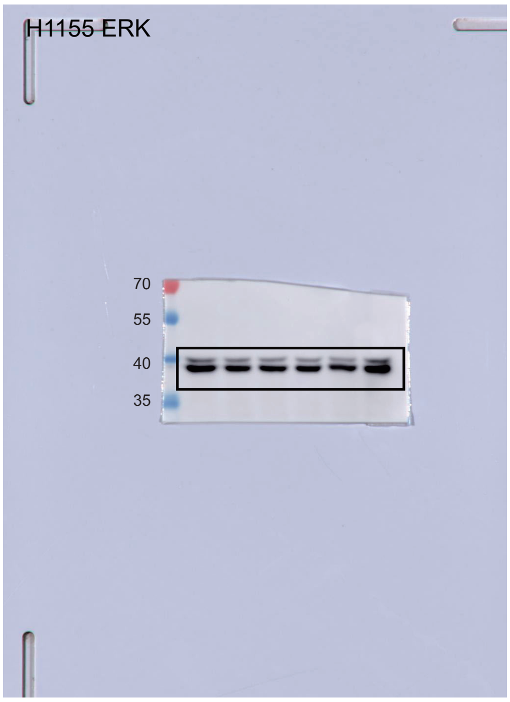

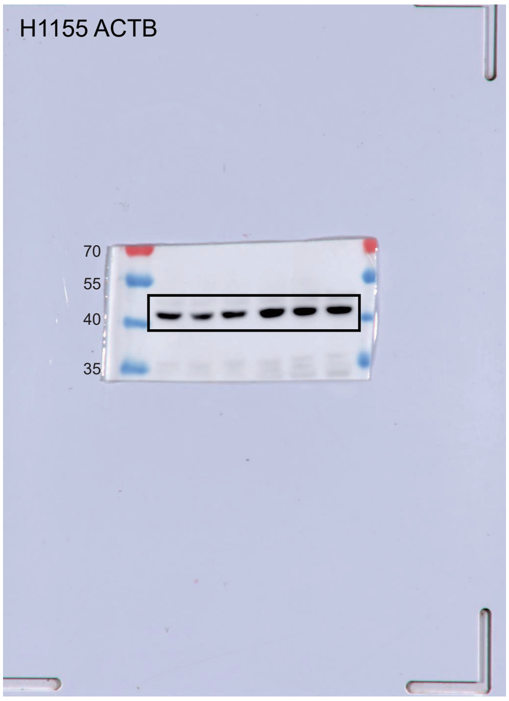

Full unedited gels for Figure S8

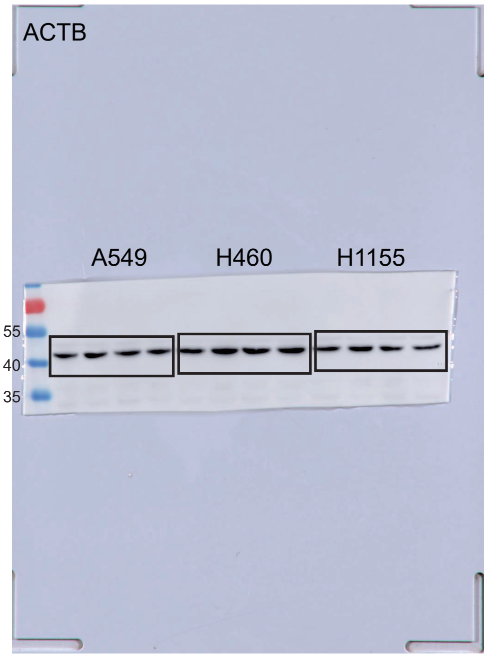

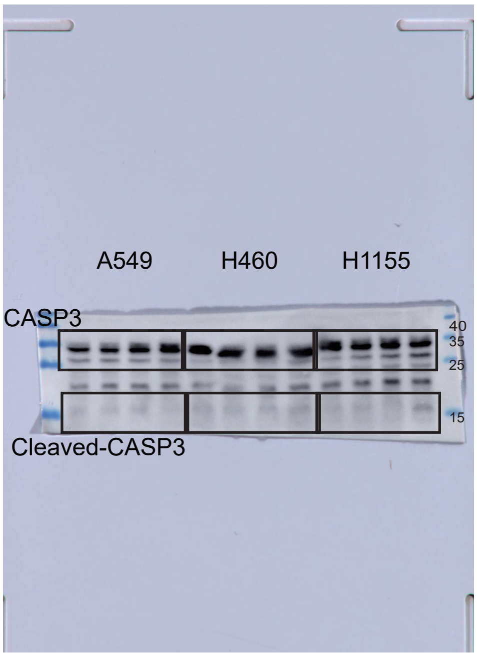

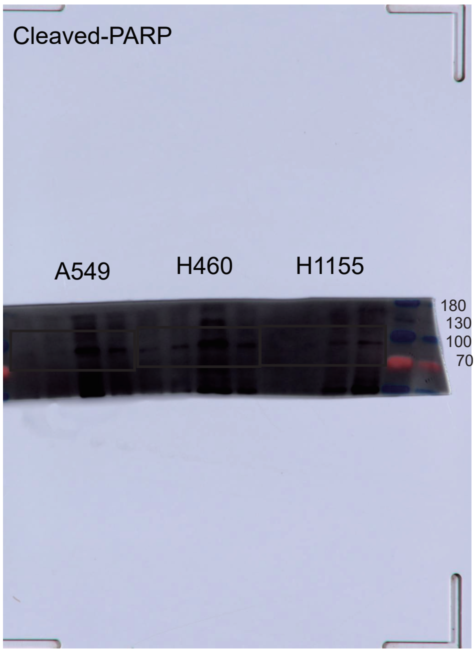

Full unedited gels for Figure S13A

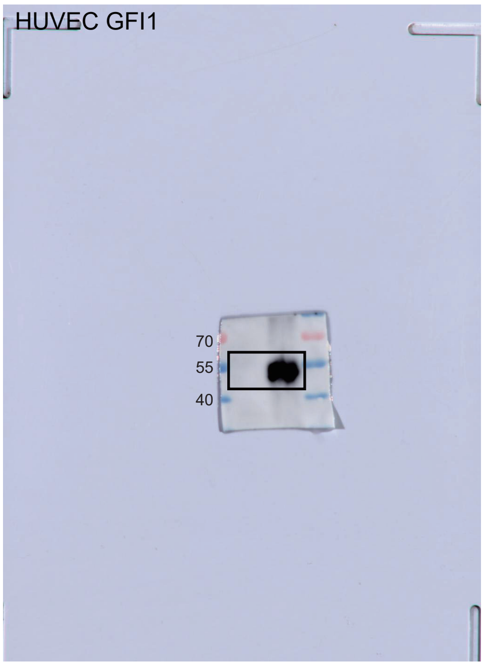

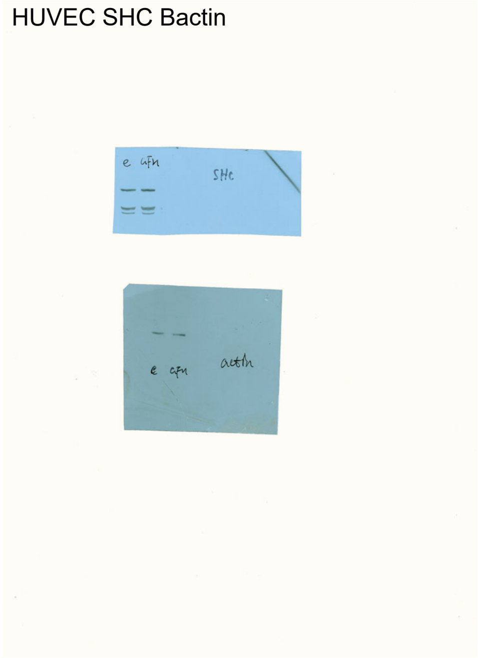

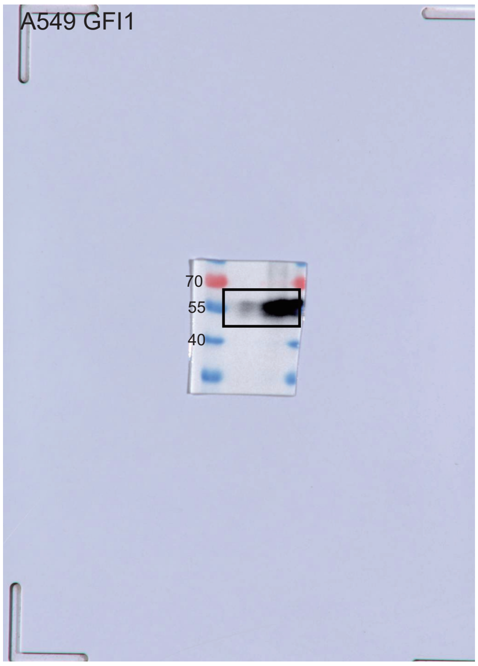

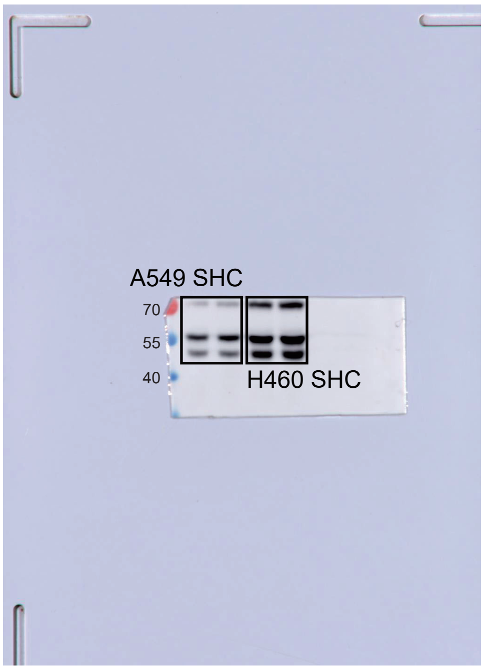

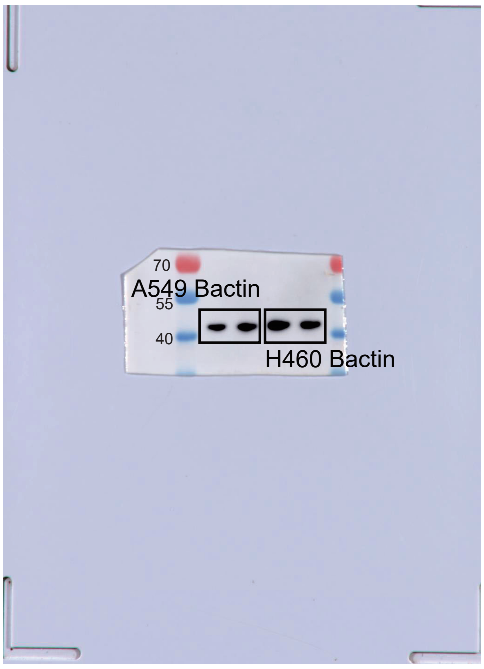

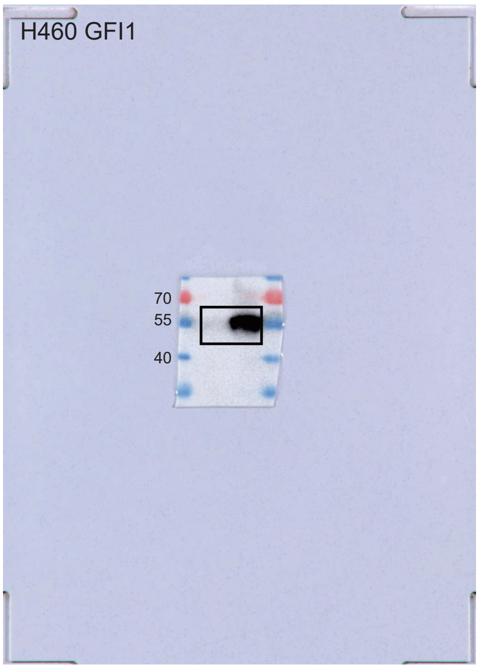

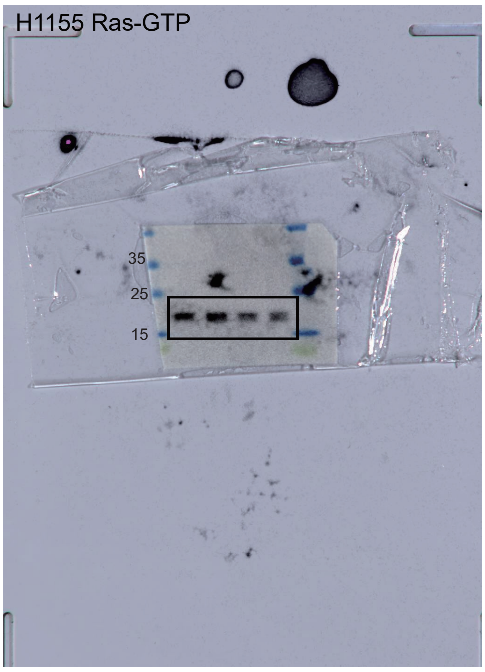

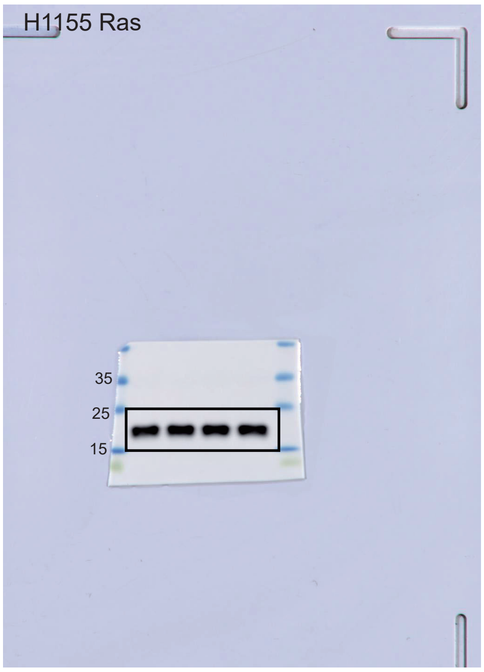

Full unedited gels for Figure S13B

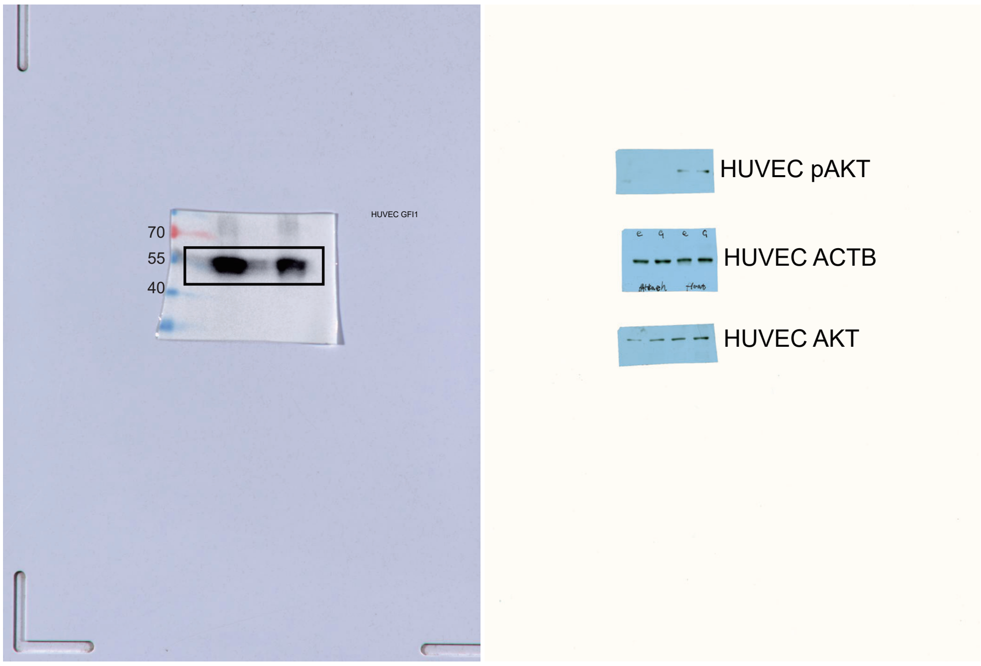

Full unedited gels for Figure S13C

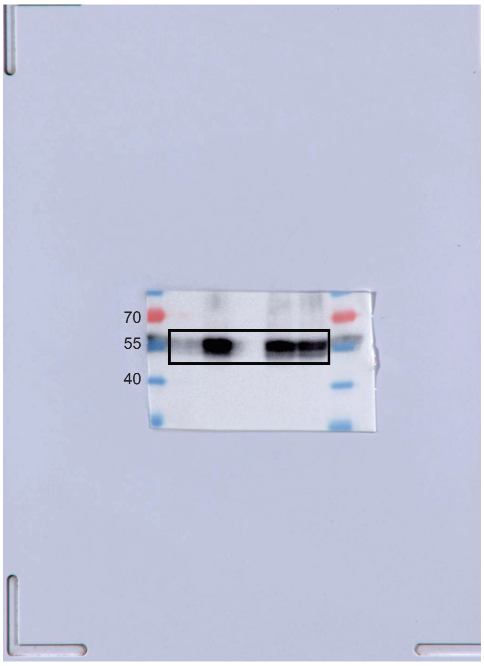

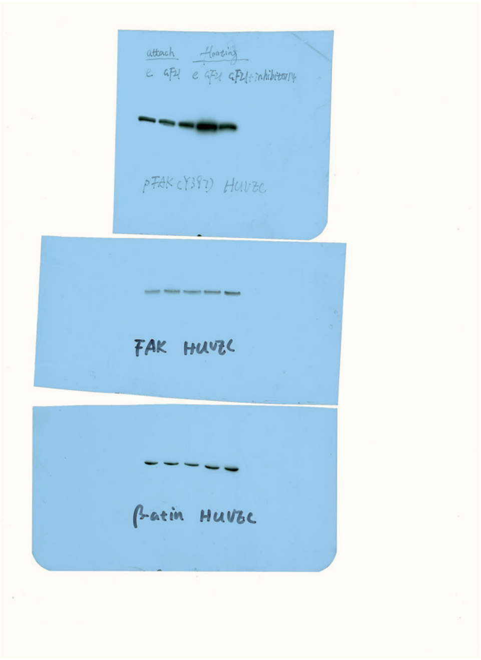

Full unedited gels for Figure S13E

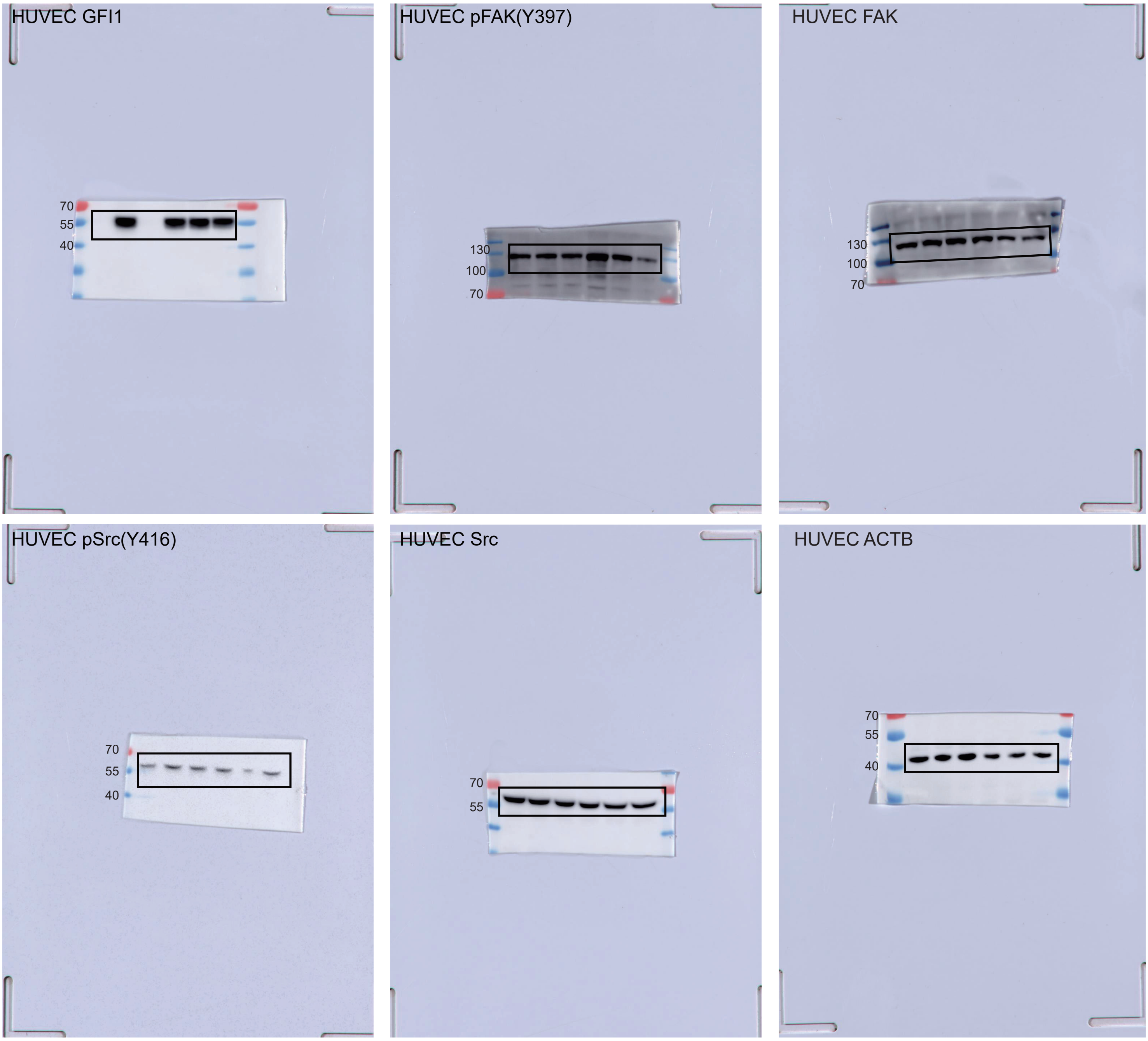

Full unedited gels for Figure S15A

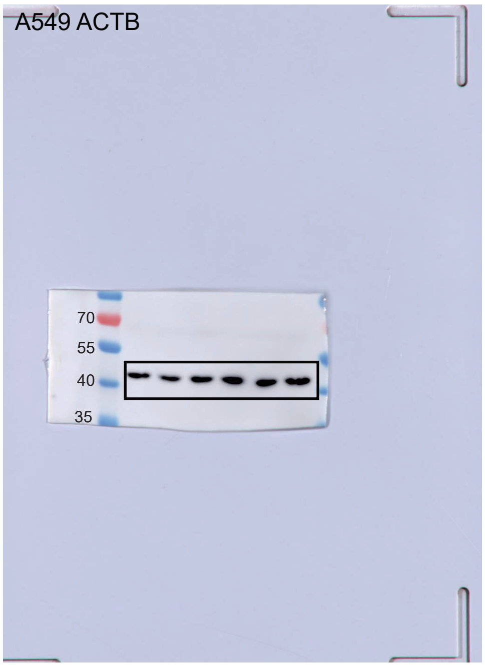

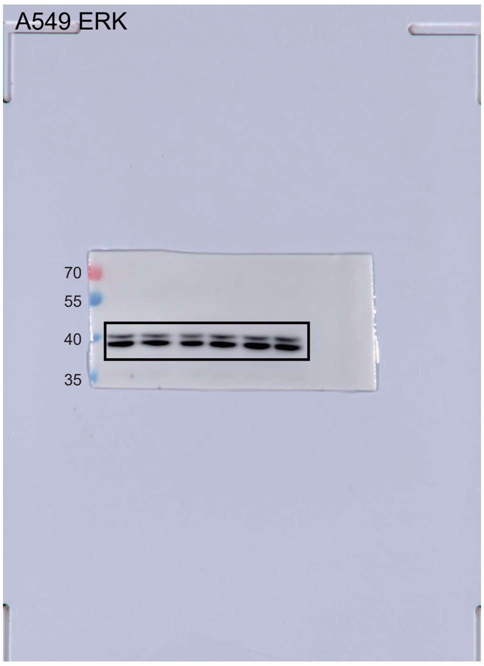

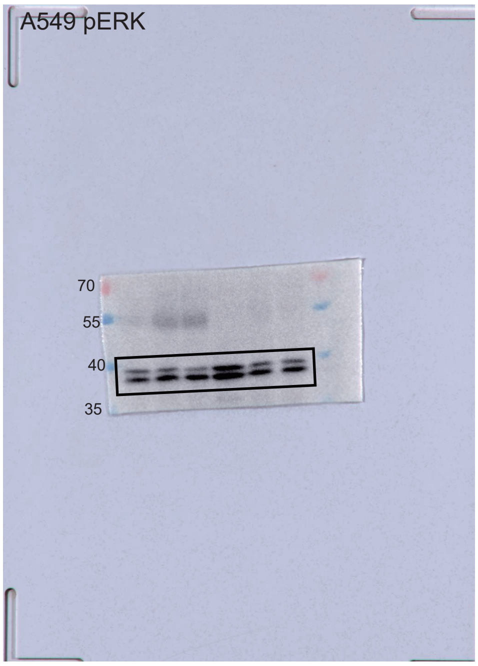

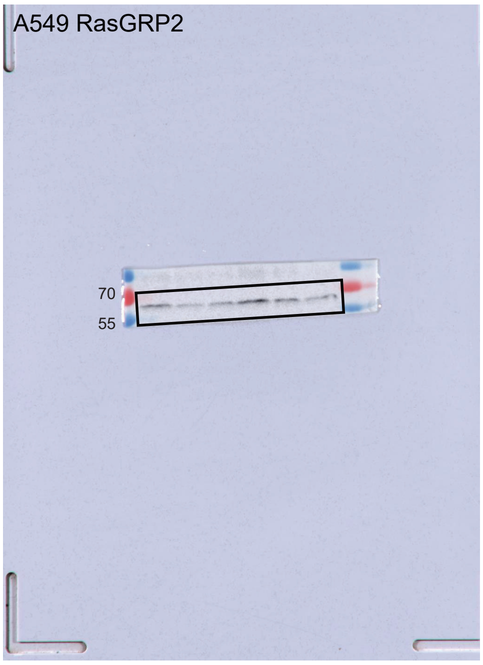

Supplement: Supplemental data [file jci-132-149551-s062.pdf]
